# Regional Climate Sensitivity of Climate Extremes in CMIP6 Versus CMIP5 Multimodel Ensembles

**DOI:** 10.1029/2019EF001474

**Published:** 2020-09-20

**Authors:** Sonia I. Seneviratne, Mathias Hauser

**Affiliations:** ^1^ Institute for Atmospheric and Climate Science, Department of Environmental Systems Science ETH Zurich Zurich Switzerland

**Keywords:** CMIP6, CMIP5, regional climate sensitivity, climate extremes, climate models, climate projections

## Abstract

We analyze projected changes in climate extremes (extreme temperatures and heavy precipitation) in the multimodel ensembles of the fifth and sixth Coupled Model Intercomparison Projects (CMIP5 and CMIP6). The results reveal close similarity between both ensembles in the regional climate sensitivity of the projected multimodel mean changes in climate extremes, that is, their projected changes as a function of global warming. This stands in contrast to widely reported divergences in global (transient and equilibrium) climate sensitivity in the two multimodel ensembles. Some exceptions include higher warming in the South America monsoon region, lower warming in Southern Asia and Central Africa, and higher increases in heavy precipitation in Western Africa and the Sahel region in the CMIP6 ensemble. The multimodel spread in regional climate sensitivity is found to be large in both ensembles. In particular, it contributes more to intermodel spread in projected regional climate extremes compared with the intermodel spread in global climate sensitivity in CMIP6. Our results highlight the need to consider regional climate sensitivity as a distinct feature of Earth system models and a key determinant of projected regional impacts, which is largely independent of the models' response in global climate sensitivity.

## Key Points

- Changes in climate extremes as a function of global warming are quasilinear and determine a “regional climate sensitivity” in CMIP5 and CMIP6
- The regional climate sensitivity of climate extremes is found to be very similar in CMIP5 and CMIP6, unlike global climate sensitivity
- Model spread in regional climate sensitivity in CMIP6 contributes more to uncertainty of projected extremes than global climate sensitivity

## 1 Introduction

Extensive literature is available on the representation of global (transient and equilibrium) climate sensitivity in different generations of Earth system models (ESMs) (Forster et al., 2019; Knutti & Hegerl, 2008; Otto et al., 2013). Global climate sensitivity refers to the response of global mean temperature to changes in CO_2_ concentrations, either in transient simulations (also termed “global transient response”) or at climate equilibrium. These global features are regularly reported prominently in first analyses of new multimodel experiments, such as when the first simulations of the sixth phase of the Coupled Model Intercomparison Project (CMIP6, Eyring et al., 2016) were released in mid‐2019 (Forster et al., 2019; Sellar et al., 2019; Swart et al., 2019).

**Figure 1:**
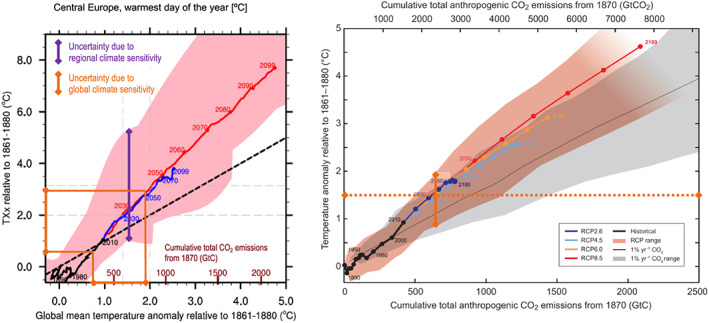
(left) Regional climate sensitivity of changes in annual hottest daytime temperature (TXx) in Central Europe (CEU, see Supporting Information, Figure S1) in the fifth phase of the Coupled Model Intercomparison Project (CMIP5), derived from empirical scaling relationships (ESRs) as a function of global warming (source: Seneviratne et al. 2016). The blue (red) line indicates the multimodel mean of the RCP4.5 (RCP8.5) CMIP5 simulations. The spread in the ESR response at +1.5°C of global warming is shown in violet and spans approximately 4°C (from 0.91°C to 5.06°C, see Table S1). The regional CEU spread in TXx resulting from the uncertainty in global (transient) climate sensitivity (also termed global transient response) based on the IPCC AR5 (see right‐hand panel) is indicated in dark orange and spans about 2.5°C. (right) Global mean surface temperature increases as a function of cumulative total global CO_2_ emissions from various lines of evidence (source: Figure SPM.10 of IPCC AR5 working group 1 report (IPCC, 2013), based on the CMIP5 ensemble; modification: dark orange lines indicate the response at +1.5°C of global warming and the respective spread in the CMIP5 RCP simulations). The figure shows multimodel results from a hierarchy of climate‐carbon cycle models for each RCP until 2100 with colored lines and decadal means (dots). For more details, see referenced publications.

However, the regional climate sensitivity in ESMs, that is, their regional responses as a function of global warming (Seneviratne et al., 2016, 2018; Wartenburger et al., 2017), can be just as important, if not more. The Paris Agreement has defined climate targets for global warming limits, and thus, a main question for society is how changes in extremes would respond at these different global warming levels. For some extremes and regions, these responses can show a large spread: in CMIP5, these were found in some cases to be much larger than the uncertainty resulting from the intermodel spread in global climate sensitivity (Seneviratne et al., 2016, see Figure 1).

Here, we investigate the regional climate sensitivity of climate extremes in the CMIP6 (Eyring et al., 2016) and CMIP5 (Taylor et al., 2012) multimodel ensembles. While first reports have investigated differences in global (transient or equilibrium) climate sensitivity in the CMIP6 ensemble (Forster et al., 2019), analyses of differences in regional climate sensitivity in the two ensembles are still lacking to our knowledge.

## 2 Methods and Data

We analyze three extreme indices defined by the expert group on Climate Change Detection and Indices (ETCCDI) (Karl et al., 1999; Peterson et al., 2001), namely, the annual hottest daytime temperature (TXx), coldest nighttime temperature (TNn), and the annual maximum 1‐day precipitation (Rx1day). The regional responses in these extremes as a function of global warming are presented as global maps of the indices at given warming levels (“warming‐level maps”) and as changes in the analyzed indices as function of mean global warming based on transient simulations (“scaling plots” or “empirical scaling relationships” [ESR] Seneviratne et al., 2016, 2018; Wartenburger et al., 2017). To ensure comparability between CMIP5 and CMIP6, we process all data in the same  way.

### 2.1 CMIP5 and CMIP6 Data

All data used stem from the CMIP5 (Taylor et al., 2012) and CMIP6 (Eyring et al., 2016) repositories. For CMIP5, we combine historical simulations (1850 to 2005) with *representative concentration pathway* (RCP) projections running from 2006 to 2100 (Meinshausen et al., 2011), using all four RCPs (RCP2.6, RCP4.5, RCP6.0, and RCP8.5). In CMIP6, we combine the historical simulations (1850 to 2015) with the *shared socioeconomic pathways* (SSPs) projections (O'Neill et al., 2016) for the years 2016 to 2100. Here, we restrict ourselves to SSP1‐1.9, SSP1‐2.6, SSP2‐4.5, SSP3‐7.0, and SSP5‐8.5. This selection of SSPs includes all Tier 1 scenarios (SSP1‐2.6, SSP2‐4.5, SSP3‐7.0, and SSP5‐8.5) and additionally the scenario most consistent with a stabilisation at +1.5°C at the end of the 21st century as aimed in the Paris Agreement (SSP1‐1.9 O'Neill et al., 2016). In order to be used, models must (i) provide the relevant variables, (ii) be run from 1850 to 2100, and (iii) not have duplicate time steps or missing time steps. For each model and scenario, we only consider the first ensemble member and weigh all models equally.

We explicitly choose not to apply any model selection or weighting, although there are dependencies across the models (e.g., Knutti et al., 2013). Our main aim is to illustrate the similarities and differences between the CMIP5 and CMIP6 ensembles. Applying, for example, an observation‐based emergent constraint on the ensembles would likely decrease their differences, irrespectively of the underlying signal. Further, we only consider one ensemble member per model so as not to overemphasize models that submitted a large number of ensemble members.

Global and regional mean temperatures are derived from surface air temperature (‘tas’). TXx and TNn are derived from daily maximum (‘tasmax’) and daily minimum (‘tasmin’) surface air temperatures, respectively. Rx1day is derived from daily precipitation data (‘pr’). Global means and regional means are calculated from the original grid resolution; for the maps, the data are aggregated to a common 2.5° x 2.5° grid, using second‐order conservative remapping (Jones, 1999).

### 2.2 Global Warming Levels

The years corresponding to the given global warming levels are determined as the first 20‐year period where the global‐mean annual‐mean surface air temperature (Tglob) exceeds the targeted global mean temperature relative to 1850 to 1900 (on average, over that 20‐year period; see below). Here, we present results for global warming levels of +1.5°C, +2.0°C, and +4.0°C. The period for each warming level, model, and scenario is calculated as follows (Hauser et al., 2019):

1. Calculate latitude‐weighted global‐mean annual‐mean temperature
2. Concatenate historical data and projections (constrained to the historical/ future time period)
3. Subtract the mean of the climatological period (1850 to 1900)
4. Calculate 20‐year centered running mean
5. Find the first year in which the running mean exceeds the desired warming level (“central year”). From this, the period starts 10 years before the “central year” and ends 9 years later.

This is done for all RCPs/ SSPs. This means that each model can contribute more than one data point for a given warming level (e.g., once for RCP6.0 and once for RCP8.5). We thus have several simulations from the same model (but for different emissions scenarios) which are used in the statistics. This may slightly bias the results if the different runs by the same models are correlated. However, we show that this is not the case (for TXx; see section 3.1). We choose to mix the scenarios to use all lines of evidence and to include as many models as possible. This also builds on the finding that in CMIP5, many variables scale well with the underlying global mean temperature independently of the forcing scenario (Seneviratne et al., 2016, 2018; Wartenburger et al., 2017). Further, there are more models available to derive climate conditions at a global warming of +1.5°C than at +4°C (see section 3.1).

### 2.3 Scaling of Extreme Indices with Global Mean Temperature

For the warming‐level maps, we make use of the above‐calculated warming‐level periods. For each model, scenario, and index, we calculate the anomaly with respect to the 1850–1900 time frame.

Then, we select the 20 years when a certain warming is reached and compute the climate model's mean response over that time frame. We then average these responses over all models and scenarios. Significance of the difference between the CMIP5 and CMIP6 ensembles is tested with a Wilcoxon Mann‐Whitney *U* test (Wilks, 2011). To account for the large number of conducted tests, we apply the approach of Benjamini and Hochberg (1995), using a global *p* value of 5 %.

For the scaling plots, we follow a similar procedure as in Seneviratne et al. (2016) and Wartenburger et al. (2017), using ESRs (Seneviratne et al., 2018; IPCC, 2018). First, regional means are calculated for the 43 currently proposed AR6 regions (see Figure S1 Iturbide et al., 2020), the global ocean and the global land. Then, we calculate a centered 20‐year running mean for all available models for each year for Tglob and the extreme index. The data are subsequently grouped according to Tglob in bins of 0.5°C width and the multimodel mean; the 5th and 95th percentiles are shown.

### 2.4 Global Temperature of Emergence

We are not only interested in how regional extreme indices scale with global mean temperature but also in identifying the global mean temperature level at which their signal emerges from internal variability. This “global temperature of emergence” is calculated from the anomalies of the extreme indices with respect to 1850 to 1900. The concept is similar to that of the “time of emergence” (e.g., Hawkins & Sutton, 2012), but instead of determining the time frame at which a signal emerges from the noise, the reference is the global warming level at which the signal emerges (see also Kirchmeier‐Young et al., 2019). We follow the procedure described in section 2.2 to determine 20‐year slices when a certain warming level is reached. Using these 20 values, we test significance with a two‐sided Wilcoxon test (Wilks, 2011). This is done for all temperature levels between 0.1°C and 4.0°C in steps of 0.1°C for each model and scenario individually. Again, the approach of Benjamini and Hochberg (1995) is applied, using a global *p* value of 5 %. The temperature of emergence is then determined as the lowest global mean temperature at which the signal is significant both at that level as well as at all subsequent tested temperature levels. The analyses are done for the individual models of the CMIP5 and CMIP6 ensembles, and the multimodel mean values are displayed.

## 3 Results and Discussion

### 3.1 Regional Climate Sensitivity of Extremes: Responses at Different Global Warming Levels

**Figure 2:**
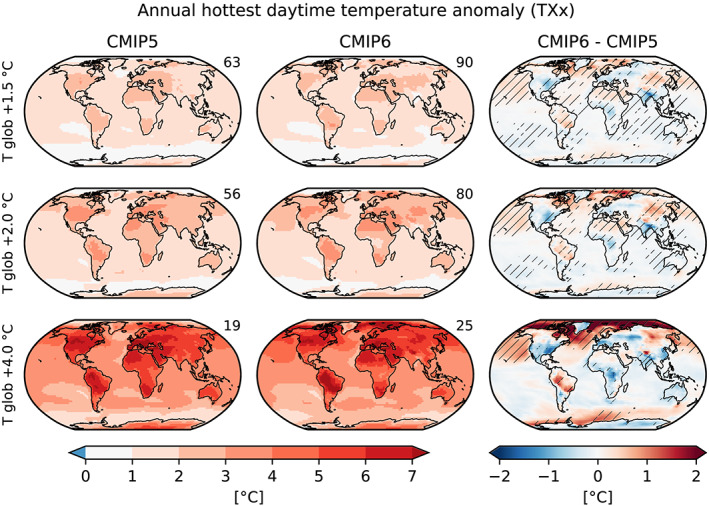
Anomalies in the annual hottest daytime temperature (TXx) compared with preindustrial (1850–1900) conditions for different global warming levels (rows) in CMIP5 (left column), CMIP6 (center column), and the CMIP6‐CMIP5 differences (right column). Statistically significant differences are hatched. The number in the top right corner of the panels indicate the number of ensemble members used.

Figure 2 displays the projected changes in TXx at +1.5°C, +2.0°C, and +4.0°C of global warming in the CMIP5 and CMIP6 multimodel ensembles, as well as the respective differences between the two multimodel means. Interestingly, the maps of regional responses at the different global warming levels are found to be very similar for the CMIP5 versus CMIP6 multimodel means. This is different to the global warming response in the two ensembles, which was shown to substantially differ for the equilibrium climate sensitivity (e.g., Forster et al., 2019). We note, however, that we consider a relatively large ensemble of CMIP6 simulations and that while this ensemble does indeed differ from CMIP5 in its global temperature projections, the overall differences are not found to be extremely large in the case of the multimodel mean (see Figure S2).

Although the most striking result in Figure 2 is the similarity of the TXx regional climate sensitivity responses in CMIP6 and CMIP5, the difference plots (right panels) show that some differences are nonetheless statistically significant between the CMIP6 and CMIP5 responses. Regions with largest increases in regional TXx sensitivity in CMIP6 are found in the South America monsoon (SAM) region, eastern Russia, the Tibetan Plateau (TIB), and eastern Sahara (SAH). Regions with largest decreases are found in Southern Asia (SAS), Central Africa (CAF), and the Eastern United States.

Notably, more statistically significant areas between CMIP6 and CMIP5 are found for differences at lower global warming levels. This is most likely an artifact of the sample size: more models in more scenarios reach a warming of +1.5°C or +2°C than +4.0°C. Analyses at all global warming levels based only on the 19 CMIP5 models and 25 CMIP6 models that reach a warming of +4.0° C are consistent with this assumption (Figure S3), as they show similar patterns as in Figure  2 but only few grid points with statistically significant differences at the lower global warming levels. We further test if mixing different scenarios biases the result by comparing two subsets of CMIP6: (i) using only SSP5‐8.5 and (ii) using all SSPs except SSP5‐8.5 (Figure S4). Despite some differences between the two subsets, these are generally nonsignificant and smaller than differences between CMIP5 and CMIP6. Hence, mixing scenarios does not have a strong influence on the derived TXx regional climate sensitivity. However, if we used all ensemble members for all models instead of only one, the signal would likely be dominated by models with a large number of ensembles. For example, differences between the full ensemble of the CMIP6 model CanESM5 (50 members per SSP) and the CMIP6 ensemble are large and mostly significant (Figure  S5).

For TNn, the overall responses in the regional climate sensitivity at different global warming levels are also highly similar in CMIP5 and CMIP6 (Figure  S6). Some statistically significant differences are found, but these are generally of small magnitude. An exception is the response in Central Europe (CEU) which shows more regional climate sensitivity (i.e., higher regional warming) in CMIP6.

**Figure 3:**
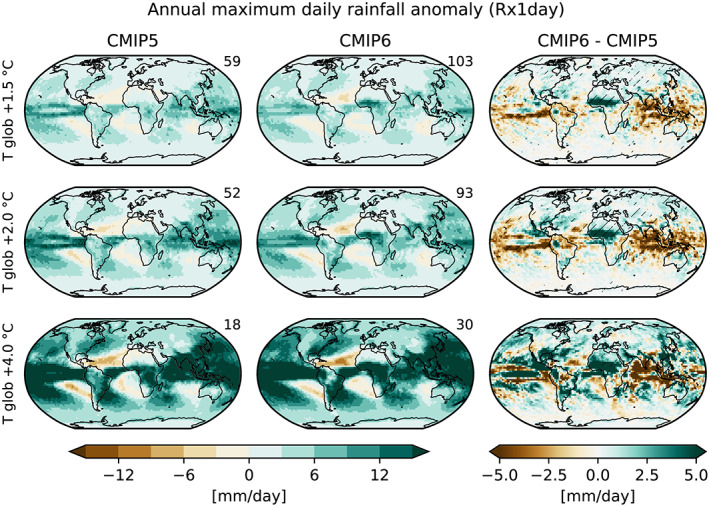
Absolute anomalies in the annual maximum daily rainfall (Rx1day) compared with preindustrial (1850–1900) conditions for different global warming levels (rows) in CMIP5 (left column), CMIP6 (center column), and the CMIP6‐CMIP5 differences (right column). Statistically significant differences are hatched.

Figure 3 provides analyses of the regional climate sensitivity of Rx1day in the CMIP5 and CMIP6 ensembles at different global warming levels (displayed as absolute differences to the preindustrial levels; for relative differences, see Figure  S7). Again, the analyses reveal little differences between the two multimodel ensembles, with the exception of the response in Western Africa (WAF) and the Sahel region (and partly also CAF). The differences in WAF and the Sahel region are found to be statistically significant, but the differences are not significant elsewhere. It would be interesting to identify the reasons for the different response in WAF and Sahel region in the two multimodel ensembles in follow‐up analyses.

### 3.2 Regional Climate Sensitivity of Extremes: Scaling Relationships

**Figure 4:**
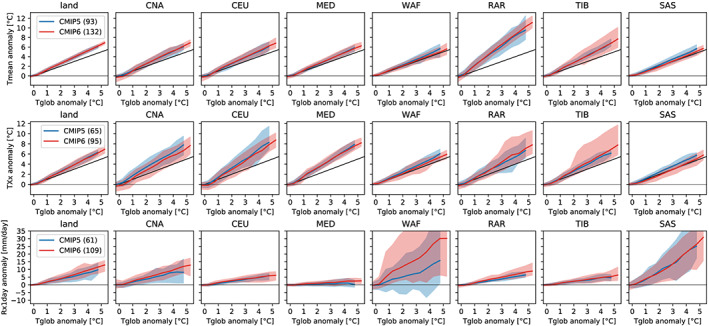
Scaling of annual mean temperature (Tmean, top row), annual hottest daytime temperature (TXx, middle row), and annual maximum daily rainfall (Rx1day, bottom row), with global mean temperature for a selection of AR6 land regions (see Supporting Information for plots for all regions). Shown are the CMIP5 and CMIP6 multimodel mean and their range (minimum to maximum value from all available ESMs) for the regions “land” (land average), central North America (CNA), Central Europe (CEU), Mediterranean (MED), Western Africa (WAF), Russian Arctic (RAR), Tibetan Plateau (TIB), and Southern Asia (SAS). See Figure S1 for a definition of the regions.

ESRs for the analyzed extreme indices in the CMIP5 and CMIP6 ensembles are provided for a set of considered Intergovernmental Panel on Climate Change (IPCC) AR6 regions (Figure S1) in the Supporting Information (Figures S8–S15). A subset of these analyses for eight regions displaying noteworthy differences between CMIP5 and CMIP6 (for TXx and Rx1day) is displayed in Figure 4, which also includes the scaling of the mean temperature (Tmean). Results for TNn are shown in the Supporting Information (Figures S12–S13). Consistent with previous analyses (Seneviratne et al., 2016; Wartenburger et al., 2017), we find a mostly linear response of the regional multimodel mean changes in mean temperature, temperature extremes, and heavy precipitation as a function of global warming.

Overall, these analyses further confirm that the regional climate sensitivity of the analyzed extreme indices is very similar in the CMIP5 and CMIP6 multimodel ensembles, with few limited exceptions (Figure 4). The scaling in Tmean is generally close to identical in CMIP5 and CMIP6 (see top row of Figure 4). This particularly applies to the multimodel mean response, while a few regions show differences in spread. This is for instance the case for the Russian Arctic region (RAR), which displays less spread in the regional climate sensitivity of Tmean in CMIP6 compared with CMIP5 (Figure 4), with the overall CMIP6 ensemble displaying very high regional mean warming sensitivity in this region (increases of about +8°C when global mean warming reaches +4°C). On the other hand, CMIP6 displays more spread in the Tmean response in the TIB compared with CMIP5 (Figure 4). Substantial differences in spread are also found in Northeast Africa (decrease in CMIP6; Figure S9).

The regional climate sensitivity of TXx as a function of global warming is also generally linear, but not identical to that of Tmean (Figure 4). This shows that regional extreme temperatures display separate features from regional mean temperatures, despite links between the two. This is particularly the case in regions with strong soil moisture feedbacks (e.g., central North America [CNA], CEU, Mediterranean [MED]), as also highlighted in Orlowsky and Seneviratne (2013), Seneviratne et al. (2016), and Vogel et al. (2017). Consistent with Figure 2, with the exception of a few regions (e.g., warming in SAM region, cooling in SAS, CNA, and CAF regions; Figure 4, Figures S10 and S11), the response of the multimodel mean regional climate sensitivity of TXx is hardly modified in CMIP6 compared with CMIP5. There are some differences in spread between CMIP5 and CMIP6 that can be identified in some regions. Regions displaying most marked changes in spread between CMIP5 and CMIP6 for TXx include CEU, the RAR, the TIB, and SAS in Figure 4, and CAF in Figure S11. These include both regions displaying less (CEU) or more (RAR, TIB, SAS, CAF) spread in CMIP6. In the case of CNA and CEU, it is noteworthy that the changes from CMIP5 to CMIP6, which show less models with high regional responses in hot extremes as function of global mean temperature (Figure 4: decreased mean response in CNA, less models with high responses in CEU), likely imply a better consistency with observations given recent analyses (Donat et al., 2018; Vogel et al., 2018). On the other hand, it is not clear if the larger spread found for the regional climate sensitivity of TXx in the RAR, TIB, SAS, or CAF regions implies an improvement or deterioration in the CMIP6 ESMs' performance. Hence, while a few differences between CMIP5 and CMIP6 can be identified in the regional climate sensitivity of TXx, the dominant feature is a strong similarity of responses between the two multimodel experiments.

In the case of Rx1day, there are also very few differences found in the regional scaling relationships derived in the CMIP5 and CMIP6 ensembles. One region with larger differences, WAF, is shown in Figure 4. It also displays a different multimodel mean response in the two ensembles, with higher increases in CMIP6. Overall, the regions displaying a different response in CMIP6 (e.g., also the MED region, Figure 4, and SAH region, Figure S14) show a moderate increase in the Rx1day regional sensitivity compared with CMIP5. These responses are consistent with the analyses from Figure 3.

### 3.3 Regional Climate Sensitivity of Extremes: Global Temperature of Emergence

**Figure 5:**
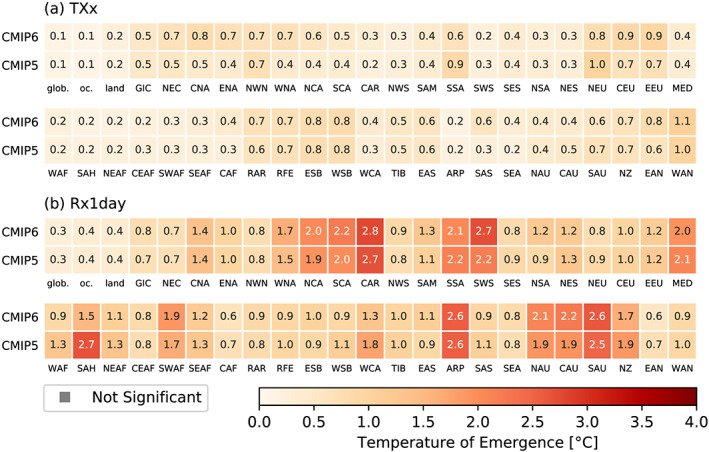
Global temperature of emergence of signal in regional climate extremes (a) TXx and (b) Rx1day for regions of Figure  S1. The colored squares indicate the multimodel mean global warming level at which a difference in response compared with preindustrial conditions (1850–1900, see section 2.4) is statistically significant. Note that the lowest tested warming level is 0.1°C.

Figure 5 displays the identified “global temperature of emergence” (section 2.4) in signals of regional climate extremes compared with preindustrial conditions (1850 to 1900). The analyses are based on 20‐year time slices of individual models and summarized as multimodel mean for TXx and Rx1day, for both CMIP5 and CMIP6 multimodel ensembles.

The results show an early emergence of regional climate signals for TXx while Rx1day requires higher temperatures for signal emergence. The TXx signal emerges at less than 0.5°C of global warming for most regions while for Rx1day, a global mean warming of more than 0.6°C is required. The regions that require the largest warming levels, however, tend to be either small, for example, the Caribbeans (CAR) or very dry, for example, the Arabian Peninsula (ARP). In line with our other results, the temperature of emergence from CMIP5 and CMIP6 are very similar. The differences between CMIP5 and CMIP6 are no larger than 0.3°C for TXx and 0.5° C for Rx1day, with the exception of the SAH. The results are qualitatively consistent with those of Kirchmeier‐Young et al. (2019) for a subset of two ESMs and based on analyses with aggregated grid cell  data.

### 3.4 Comparing the Regional Uncertainty Resulting From Regional Versus Global (Transient) Climate Sensitivity

**Figure 6:**
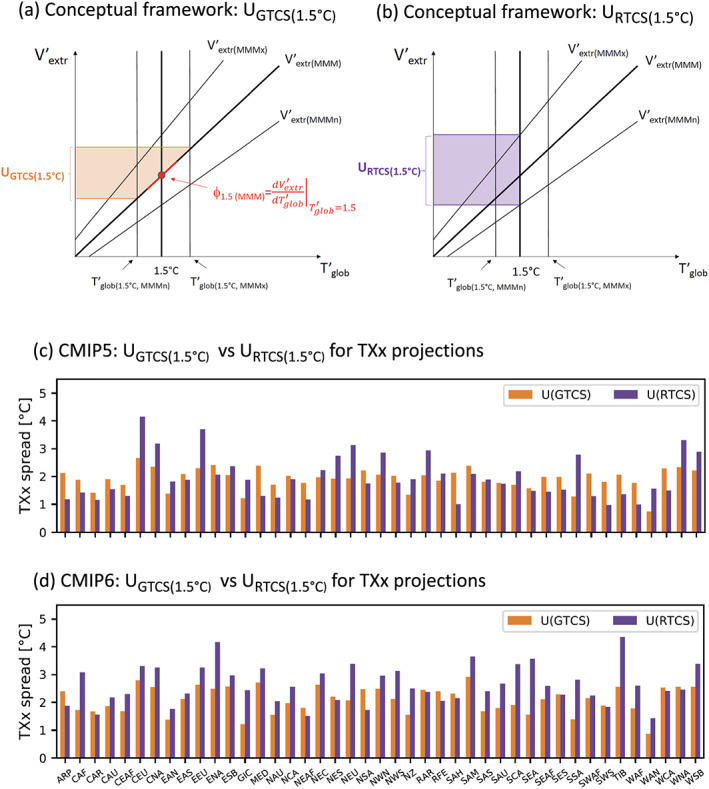
Comparison of uncertainty in projections of regional climate extremes resulting from global transient climate sensitivity *U_GTCS_* and regional transient climate sensitivity *U*_*RTCS*_ +1.5°C of global warming. (a,b) Conceptual framework for computation of *U*_*GTCS*_ (a) and *U*_*RTCS*_ (b) for anomalies in a given climate extreme *V′_extr_* as function of global warming *T′* *_glob_*; see text for details. (c,d) Regional values of *U*_*GTCS*_ (orange) and *U*_*RTCS*_ (violet) in CMIP5 (c) and CMIP6 (d). See Figure S1 for regions.

An important and yet unaddressed question is the respective contribution of the regional versus global climate sensitivity for regional changes in extremes. Indeed, while much effort has gone into constraining global climate sensitivity (Cox et al., 2018; Knutti & Hegerl, 2008; Otto et al., 2013), resulting uncertainties are mostly of relevance if they affect impacts. As many impacts are associated to regional changes in extremes (e.g., Hoegh‐Guldberg et al., 2018), it is useful to assess if the uncertainties in projected regional climate extremes are mostly resulting from uncertainties in global versus regional climate sensitivity. To compare these respective contributions, we use the following framework (Figure 6). We focus here on uncertainties in projections at +1.5°C of global warming. We thereby compare the uncertainty resulting from uncertainty in the global transient climate sensitivity, referred to as *U*_*GTCS*_, with that resulting from the uncertainty of the regional transient climate sensitivity for the considered extreme, referred to as *U*_*RTCS*_.

We assume that changes in a given climate extreme of interest *V*_*extr*_ as a function of mean global temperature *T*_*glob*_ can be approximated with a linear relationship (Figure 6), consistent with, for example, Figure 4. Hereafter, *V*_*extr*_ and *T*_*glob*_ are expressed as anomalies compared with preindustrial conditions, *V′_extr_* and *T′_glob_*, for ease of comparison with Figures 1 and 4. To compute *U*_*GTCS*_, we first determine the multimodel mean *regional climate sensitivity* $\phi_{1.5^{\circ }C(MMM)}$ of the anomaly in the considered climate extreme $V_{extr(1.5^{\circ }C,MMM)}^{\prime }$ as a function of the mean global warming $T_{glob_{\left(MMM\right)}}^{\prime }$ *at +1.5°C*:

(1) $$\phi_{1.5^{\circ }C(MMM)}={\left(\frac{\partial V_{extr_{\left(MMM\right)}}^{\prime }}{\partial T_{glob_{\left(MMM\right)}}^{\prime }}\right|}_{T_{glob(MMM)=1.5^{\circ }C}^{\prime }}$$

(2) $$\;=\frac{V_{extr_{\left(MMM\right)}}^{\prime }(T_{glob}^{\prime }=1.5)}{1.5}.$$

We then determine the uncertainty in the estimated global mean warming from the minimum and maximum response in *T′_glob_* in the CMIP5 or CMIP6 ensembles when their respective multimodel mean reaches +1.5°C of global warming. The respective values are referred to as *T′_glob_*_(1.5,*MMMn*)_ and *T′_glob_*_(1.5,*MMMx*)_. The uncertainty in regional climate extremes resulting from the uncertainty in global transient climate sensitivity (*U*_*GTCS*_) at +1.5°C, *U*_*GTCS*(1.5)_, is then estimated as follows:

(3) $$U_{GTCS(1.5)}=\phi_{1.5^{\circ }(MMM)}*(T_{glob(1.5,MMMx)}^{\prime }-T_{glob(1.5,MMMn)}^{\prime }).$$

The respective uncertainty in *V′_extr_* resulting from the regional climate sensitivity, *U*_*RTCS*(1.5)_, is estimated for the minimum and maximum responses in *V′_extr_* when *T′_glob_* reaches +1.5°C, which are referred to as $V_{extr(1.5^{\circ }C,MMMn)}^{\prime }$ and $V_{extr(1.5^{\circ }C,MMMx)}^{\prime }$, respectively:

(4) $$U_{RTCS(1.5)}=V_{extr(1.5^{\circ }C,MMMx)}^{\prime }-V_{extr(1.5^{\circ }C,MMMn).}^{\prime }$$

We display the *U*_*GTCS*(1.5)_ and *U*_*RTCS*(1.5)_ values for the regional projected changes in TXx in CMIP5 and CMIP6 in Figures 6c and  6d, respectively. Interestingly, the uncertainty resulting from the uncertainty in regional climate sensitivity (*U*_*RTCS*(1.5)_) is often found to be larger than that resulting from global climate sensitivity (*U*_*GTCS*(1.5)_) in both CMIP5 and CMIP6. In the case of CMIP6, the contribution of *U*_*RTCS*(1.5)_ is particularly large and constitutes the dominant driver of uncertainty in a majority of regions. This highlights regional climate sensitivity as a key determinant of projected uncertainty in climate extremes and the at least equal importance of constraining regional climate sensitivity and global climate sensitivity to reduce uncertainties in projections of impacts at higher global warming levels.

Note that because of the near‐linear scaling of the regional climate sensitivity of climate extremes as a function of global warming, the ratio of *U*_*RTCS*_ versus *U*_*GTCS*_ would be similar at different global warming levels if *U*_*GTCS*_ remained more or less constant independently of global warming. This assumption is, however, not fulfilled at higher global warming levels, as seen in Figure 1 (right), and when comparing Tables S2–S5. Indeed, the overall range in *T′_glob_* is approximately 1.1°C at +1.5°C of global warming in CMIP5 and CMIP6 (Tables S2–S3, SSP1‐1.9), while it spans about 2.8–3.5°C at +4°C of global warming (Tables S4–S5). Hence, the estimates derived in Figure 6 mostly apply to low‐emission scenarios compatible with the Paris Agreement (+1.5°C or well below +2°C). The values of *U*_*GTCS*_ would be about twice larger at +4°C of global warming compared with those for +1.5°C of global warming in Figure 6.

## 4 Conclusions

In this article, we have analyzed the regional climate sensitivity of climate extremes in the CMIP5 and CMIP6 multimodel ensembles. The results show that unlike recently published results showing relatively large differences in global (transient and equilibrium) climate sensitivity, the regional climate sensitivity in the analyzed climate extremes (yearly hottest days, yearly coldest nights, and yearly heaviest precipitation events) is very similar in the CMIP5 and CMIP6 ensembles. This highlights regional climate sensitivity as a distinct feature compared with global climate sensitivity.

Reasons for the different responses in regional climate sensitivity versus global climate sensitivity are likely related to the differing underlying processes driving them. Regional climate sensitivity has been shown to be strongly affected by regional land processes such as soil moisture and snow feedbacks (Orlowsky & Seneviratne, 2013; Seneviratne et al., 2016; Vogel et al., 2018), which are, however, not strongly affecting global mean temperature. It is likely that global climate sensitivity is more strongly related to ocean‐climate interactions, because the global mean temperature corresponds for two thirds to temperatures over ocean areas. It is hence a good measure of ocean warming, but it may fail to capture specifics of land warming, as also observed during the so‐called hiatus period (Seneviratne et al., 2014). Vogel et al. (2017) have shown that projected changes in temperature extremes in midlatitude regions can be decomposed in the sum of mean global warming and an additional warming associated with soil moisture feedbacks, with the two terms being close in magnitude.

The main identified discrepancies in regional climate sensitivity in CMIP6 versus CMIP5 include some higher warming of hot extremes in the SAM region, lower warming of hot extremes in CAF, less tendency for high warming of hot extremes in CEU and CNA, and higher increases in heavy precipitation in WAF and the Sahel region. It would be useful that follow‐up analyses identify the reasons for these differences, but we note that overall, the multimodel mean responses are very similar across the two model experiments, which is the main finding of this analysis.

We also compared the uncertainty in regional projections of hot temperature extremes resulting from uncertainty in the global versus regional transient climate sensitivity within the CMIP5 and CMIP6 ensembles. Interestingly, in both ensembles, the regional climate sensitivity is found to be a substantial contributor to the overall uncertainty of regional projections at approximately +1.5°C of global warming, and it dominates the overall uncertainty in a majority of regions in CMIP6. Note that this estimate includes both effects of the forced regional signal and decadal variability.

Finally, we note that the analyses are based on the CMIP6 archive as of late March 2020 and that this archive is not yet complete. It is possible that the results based on the final CMIP6 archive could be slightly different.

In conclusion, we found that regional climate sensitivity, that is, the response of regional climate variables as a function of global mean warming, is a major determinant of the regional response of climate extremes, which is at least on par with global (transient) climate sensitivity in determining uncertainty of projections. These results highlight that better constraining processes affecting regional climate sensitivity could strongly reduce uncertainty in extremes' and impacts' projections, potentially even more so than further refinements of global climate sensitivity. It will be of high relevance that more investigations focus on regional climate sensitivity as an individual quantity to assess in the evaluation of ESMs, on equal footing with global climate sensitivity.

## Supporting information



Supporting Information S1
