# Supplementary material for: Regional Climate Sensitivity of Climate Extremes in CMIP6 Versus CMIP5 Multimodel Ensembles
Source: Earths Future. 2020 Sep 20;8(9):e2019EF001474. doi: 10.1029/2019EF001474 (PMC7539979; doi:10.1029/2019EF001474)
Supplement: Supplementary file 1 — Supporting Information S1 [file EFT2-8-e2019EF001474-s001.pdf]

Supporting Information for "Regional climate sensitivity of climate extremes in CMIP6 vs CMIP5 multi-model ensembles"

Sonia I. Seneviratne[1] **and Mathias Hauser**[1]

[1]Institute for Atmospheric and Climate Science, ETH Zurich, Zurich, Switzerland

May 4, 2020

Contents of this file

1. Figures S1 to S15
2. Tables S1 to S5

Additional figures

Corresponding author: Sonia I. Seneviratne and Mathias Hauser, sonia.seneviratne@ethz.ch, mathias.hauser@env.ethz.ch

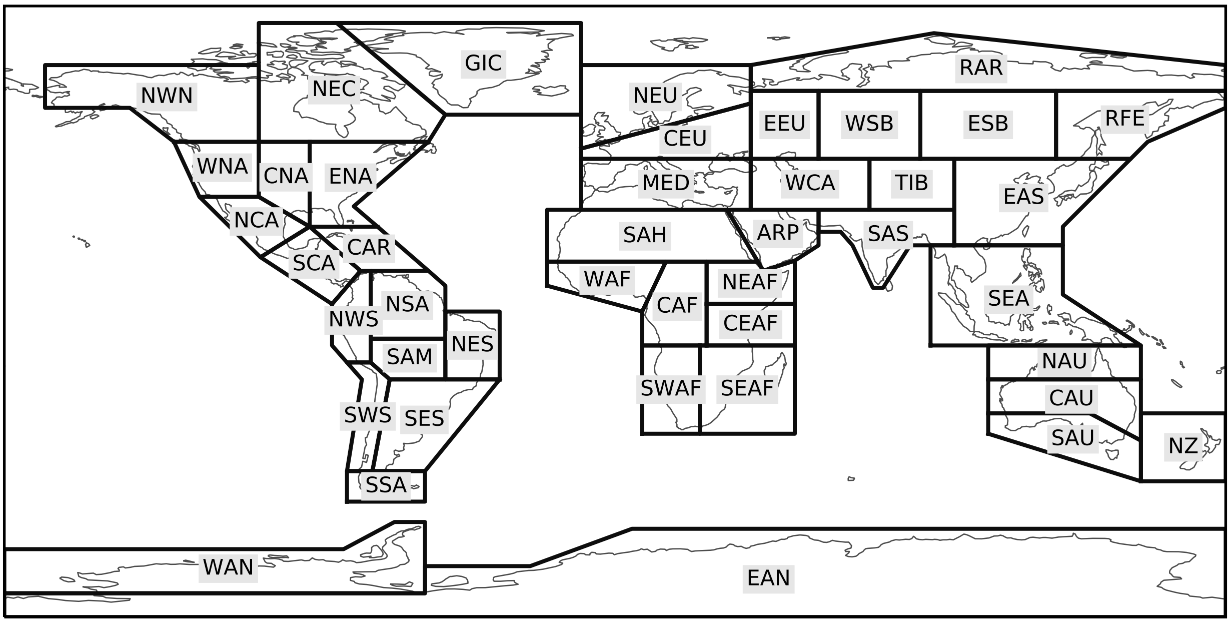

Figure S1: Regions used for analyses, based on preliminary IPCC AR6 regions (Iturbide et al., 2020): 1. GIC: Greenland/Iceland, 2. NEC: N.E. Canada, 3: CNA: C. North America, 4. ENA: E. North America, 5. NWN: NW North America, 6. WNA: Western North America, 7. NCA: Northern Central America, 8. SCA: Southern Central America, 9. CAR: Caribbean, 10. NWS: NW South America, 11. SAM: South-America Monsoon, 12. SSA: Southern South America, 13. SWS: SW South America, 14. SES: SE South America, 15. NSA: N. South America, 16. NES: NE South America, 17. NEU: N. Europe, 18. CEU: C. Europe, 19. EEU: E. Europe, 20. MED: Mediterranean, 21. WAF: W. Africa, 22. SAH: Sahara, 23. NEAF: NE Africa, 24. CEAF: Central East Africa, 25. SWAF: SW Africa, 26. SEAF: SE Africa, 27. CAF: Central Africa, 28. RAR: Russian Arctic, 29. RFE: Russian Far East, 30: ESB: E. Siberia, 31. WSB: W. Siberia, 32: WCA: W.C. Asia, 33: TIB: Tibetan Plateau, 34: EAS: E. Asia, 35: ARP: Arabian Peninsula, 36. SAS: S. Asia, 37. SEA: S.E. Asia, 38. NAU: N. Australia, 39. CAU: C. Australia, 40. SAU: S. Australia, 41. NZ: New Zealand, 42. EAN: E. Antarctica, 43. WAN: W. Antarctica.

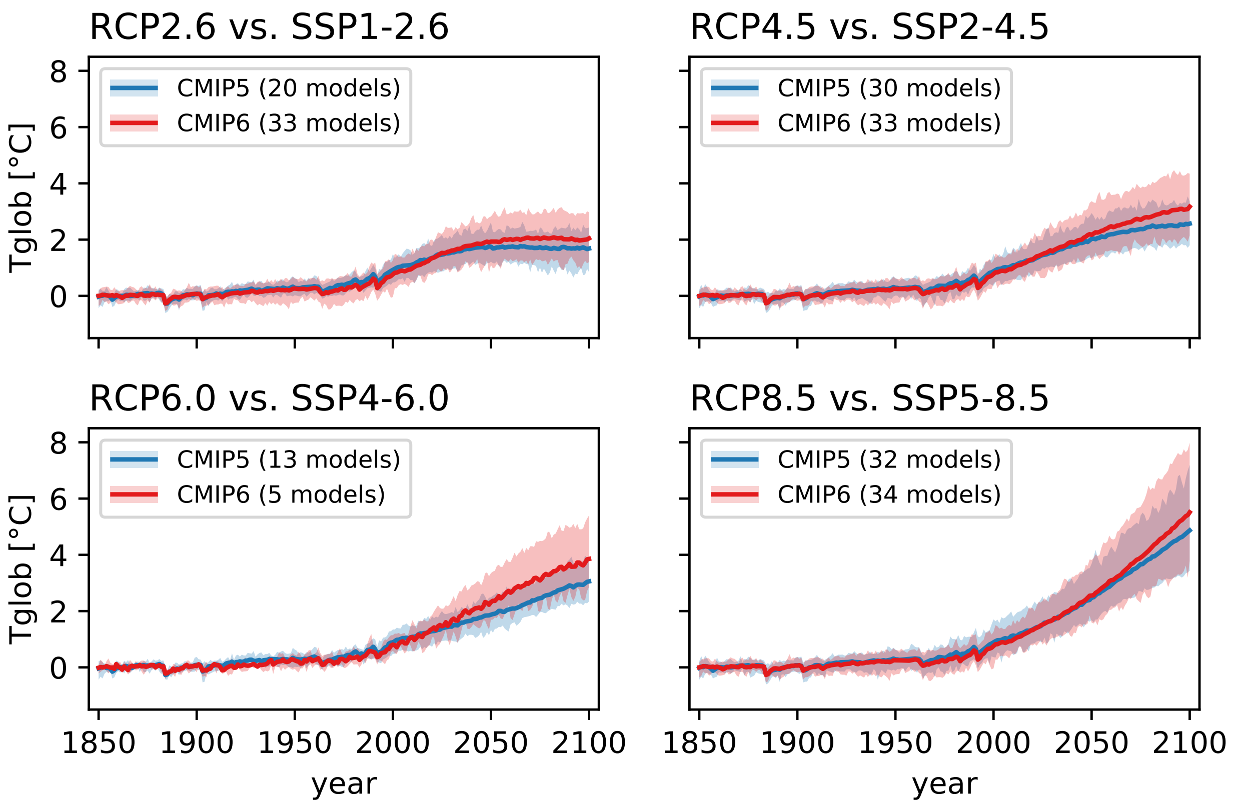

Figure S2: Time series of global mean warming in CMIP5 and CMIP6 ensembles, subdivided by scenario type.

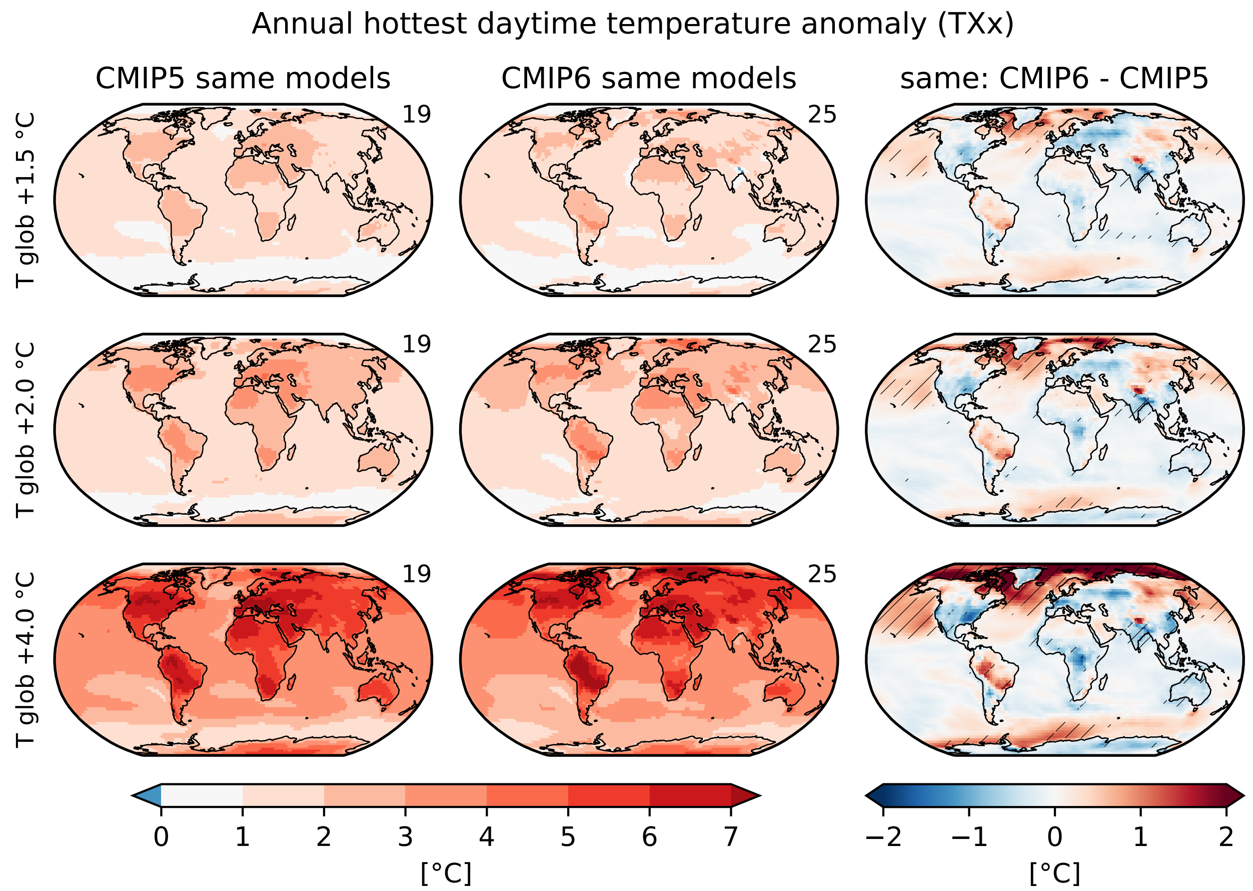

Figure S3: Anomalies in the annual hottest daytime temperature (TXx) compared to pre-industrial (1850-1900) conditions for different global warming levels (rows) in CMIP5 (left column), CMIP6 (center column), using the same models for all temperature levels, and the CMIP6-CMIP5 differences (right column). Statistically significant differences are hatched.

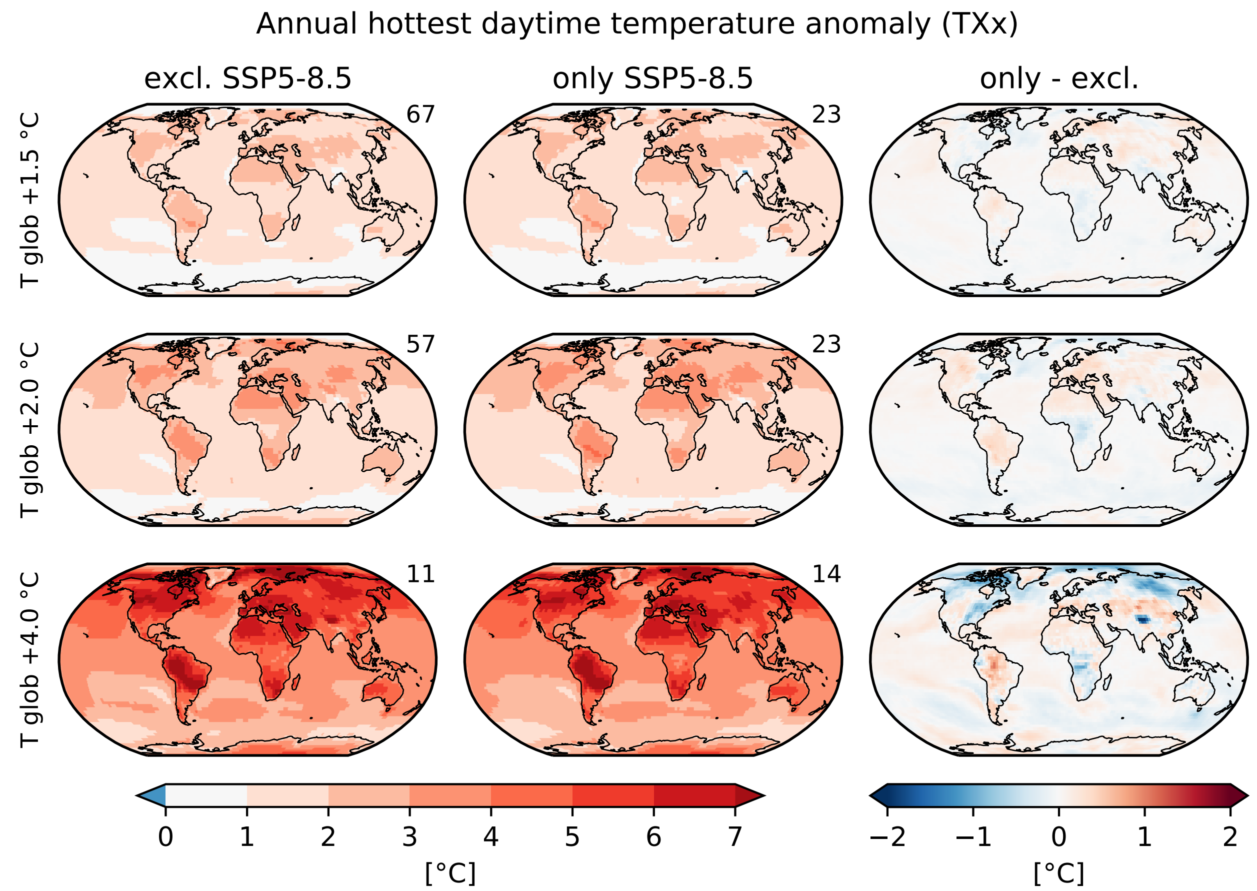

Figure S4: Anomalies in the annual hottest daytime temperature (TXx) compared to pre-industrial (1850-1900) conditions for different global warming levels (rows) using only the SSP5-8.5 scenario (left column), using all SSPs excluding (excl.) SSP5-8.5 (center column), and their differences (right column). Significant differences are hatched.

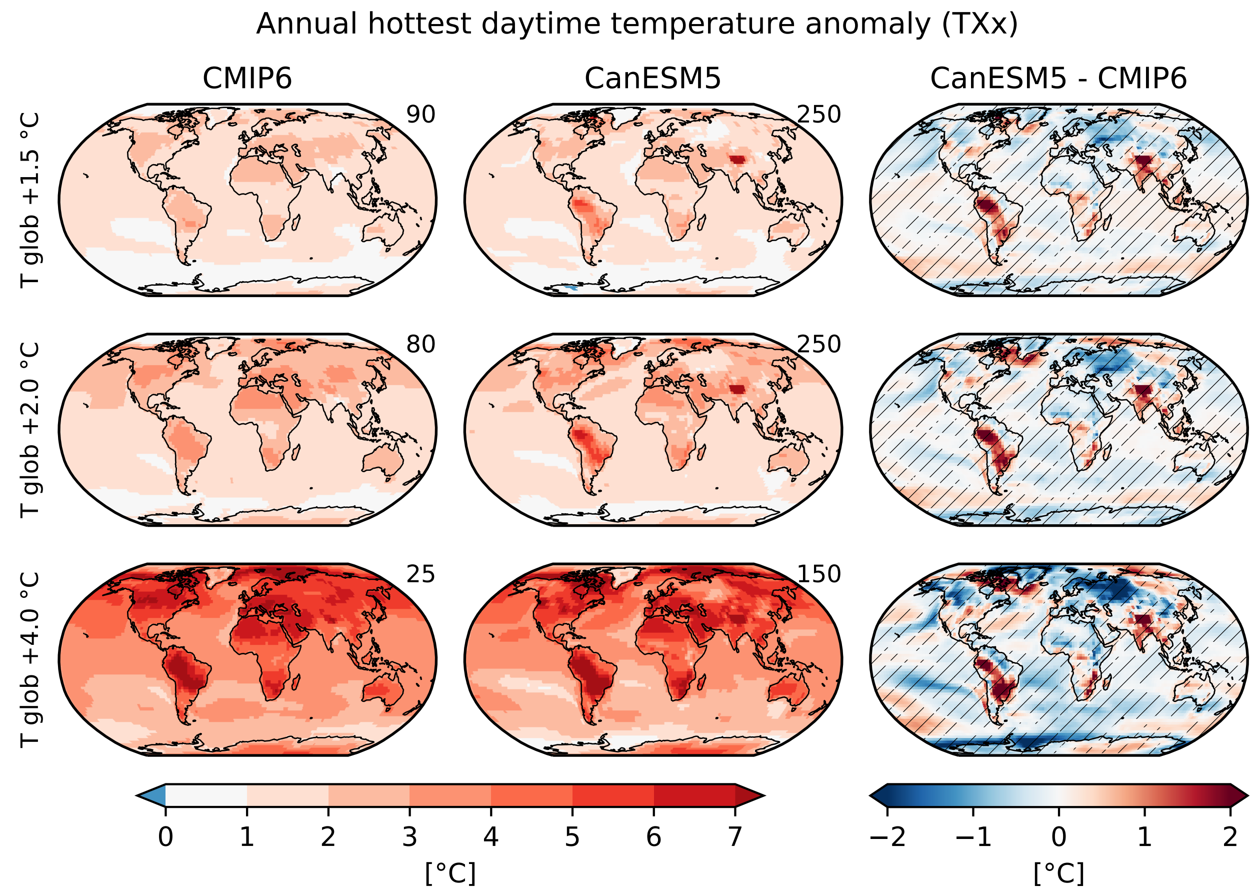

Figure S5: Anomalies in the annual hottest daytime temperature (TXx) compared to pre-industrial (1850-1900) conditions for different global warming levels (rows) using all ensemble members of the CMIP6 model CanESM5 (left column), using one ensemble member of each CMIP6 model (center column), and their differences (right column). Significant differences are hatched.

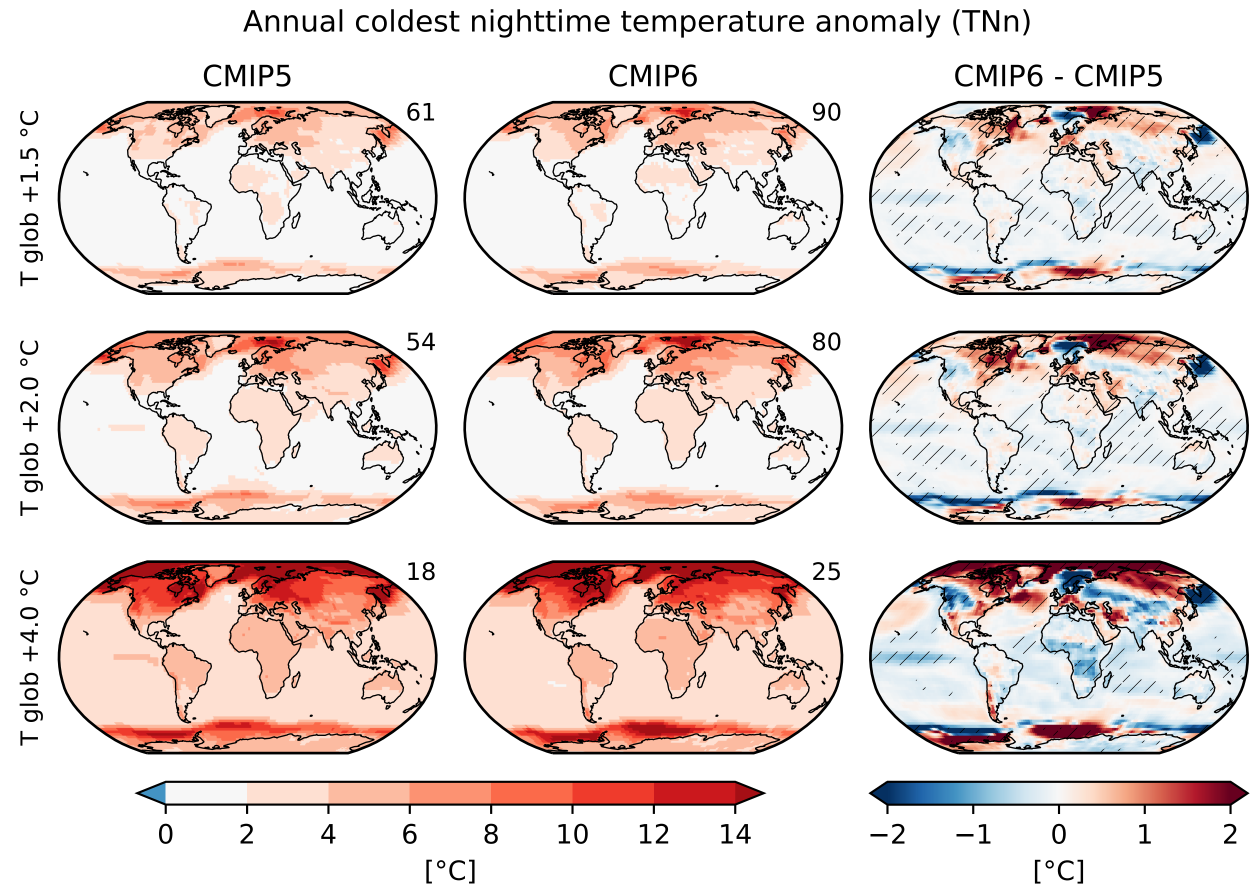

Figure S6: Anomalies in the annual coldest nighttime temperature anomaly (TNn) for different warming levels (rows) in CMIP5 (left column), CMIP6 (center column), and their difference (right column). Significant differences are hatched.

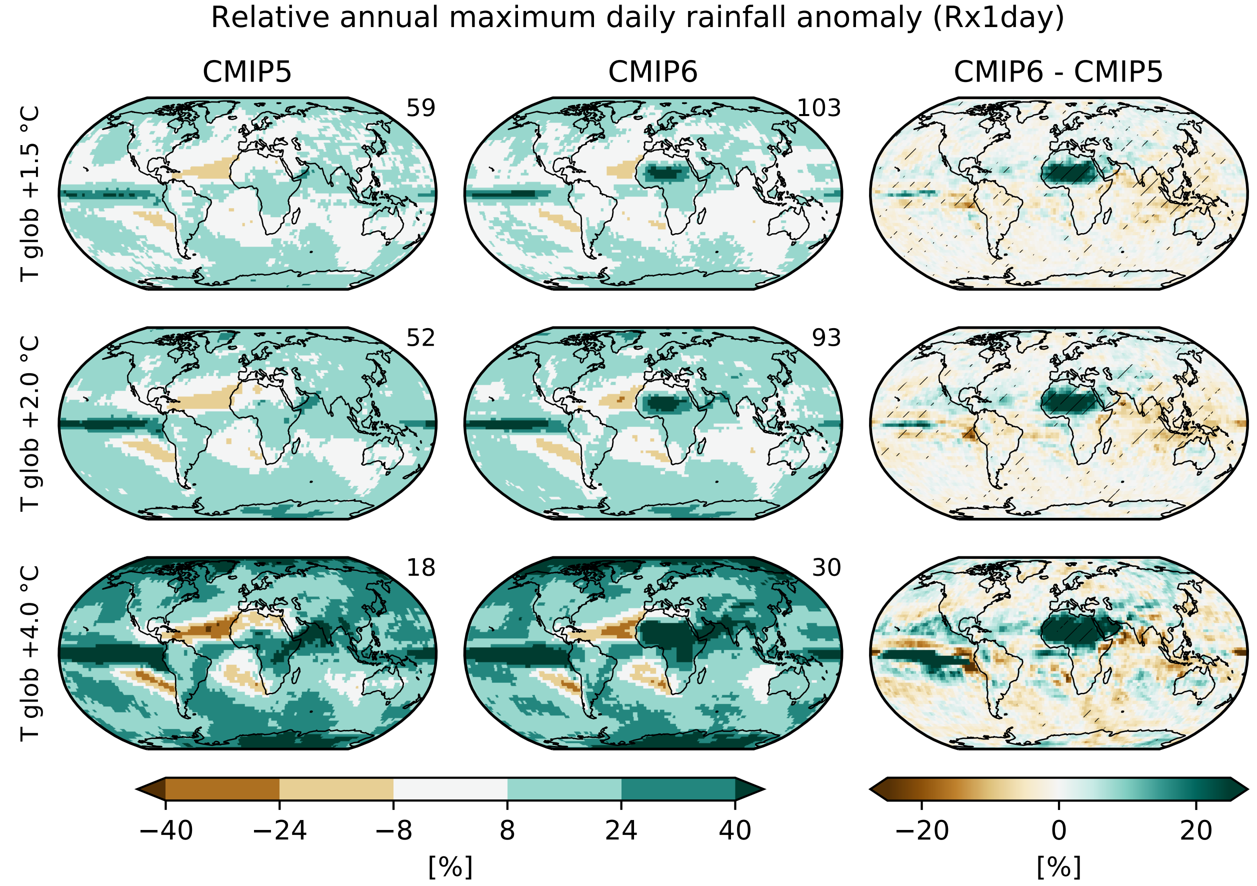

Figure S7: Relative anomalies in the annual maximum daily rainfall (Rx1day) for different warming levels (rows) in CMIP5 (left column), CMIP6 (center column), and their differences (right column). Statistically significant differences are hatched.

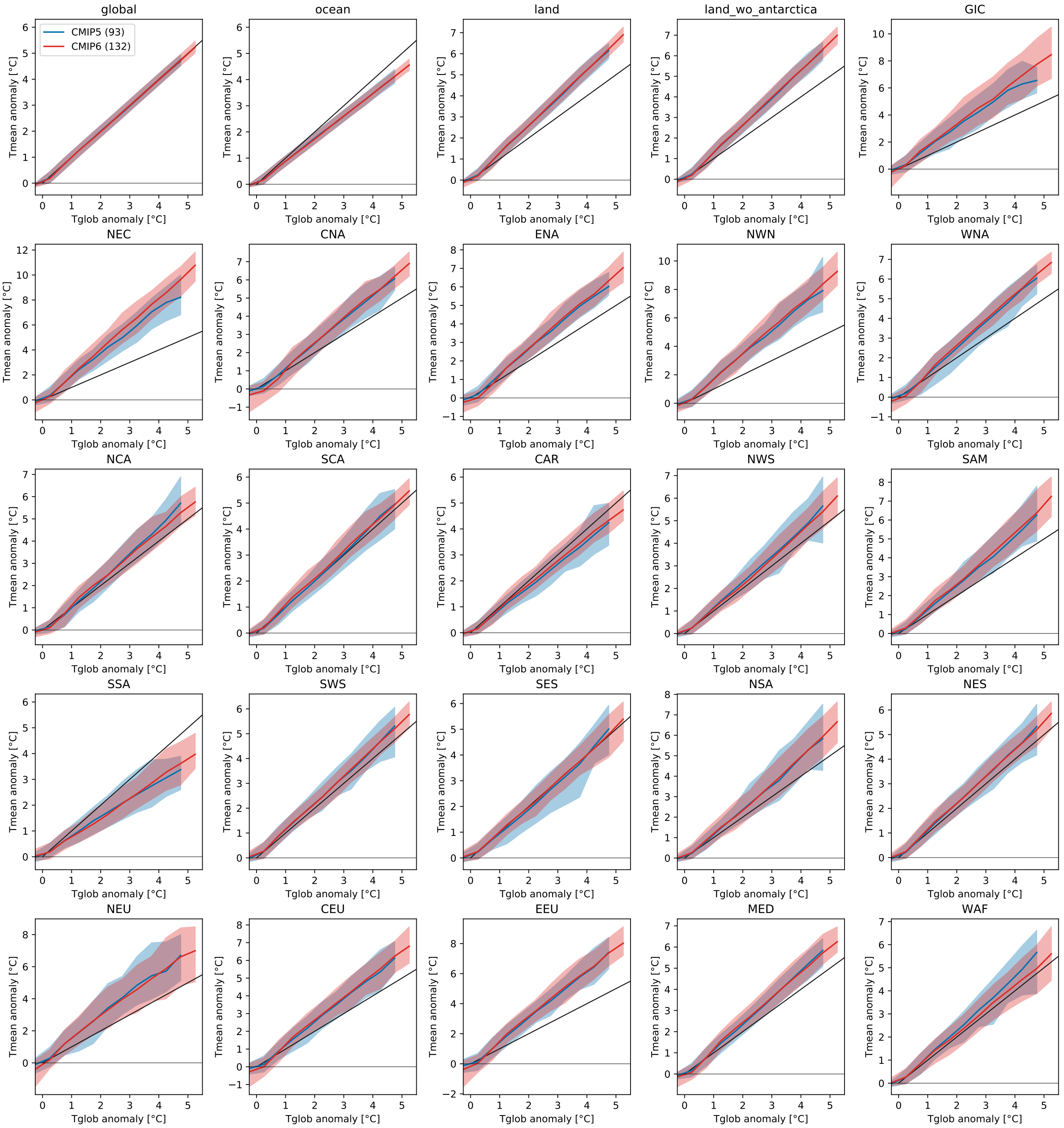

Figure S8: Scaling of annual mean temperature (Tmean) with global mean temperature for the AR6 land regions. Shown is the CMIP5 and CMIP6 multi model mean and their range, for the global mean, ocean mean, land mean, land mean except Antarctica, and regions 0 to 21 of the AR6 land regions.

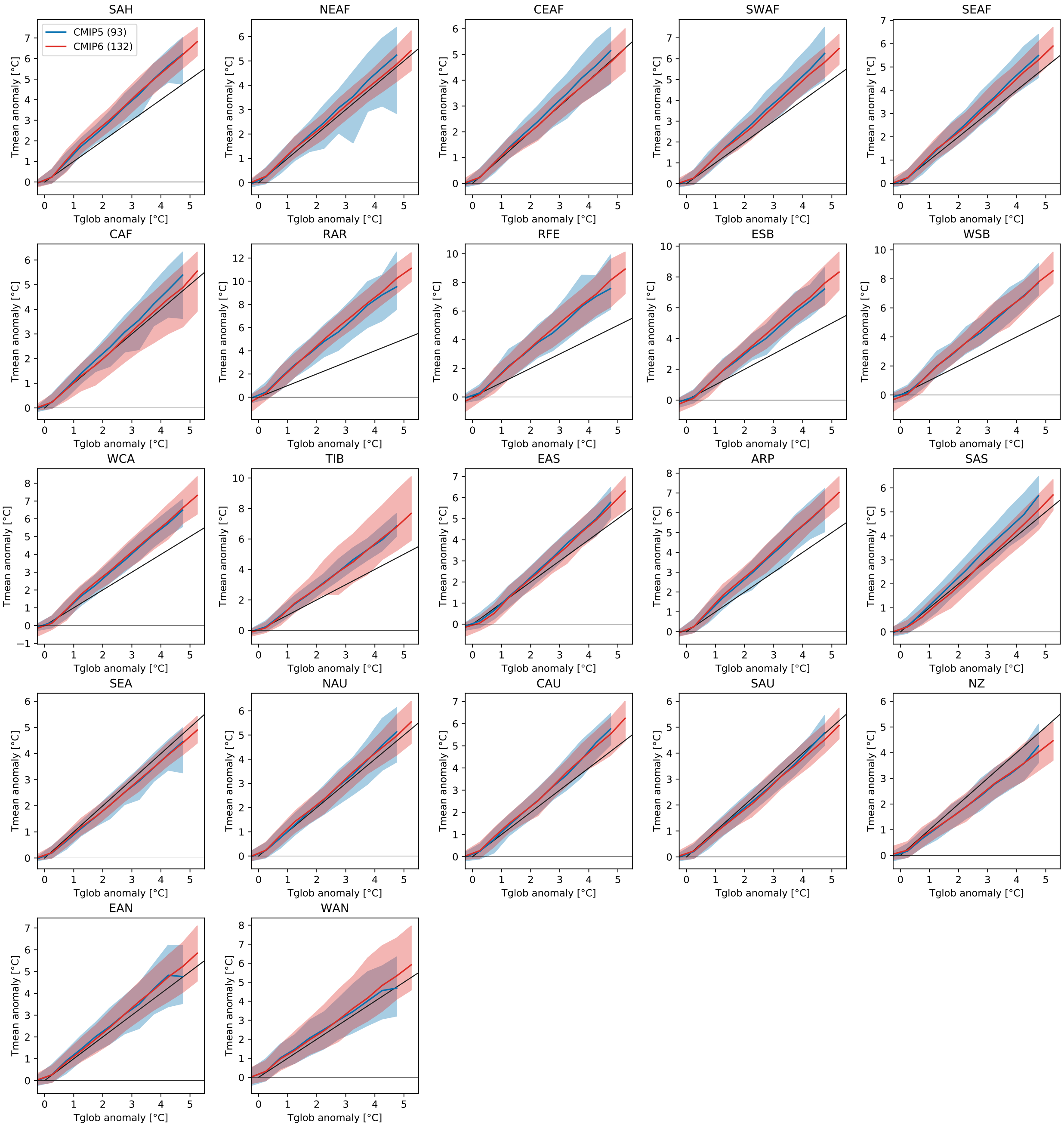

Figure S9: As Figure S8 for regions 22 to 43.

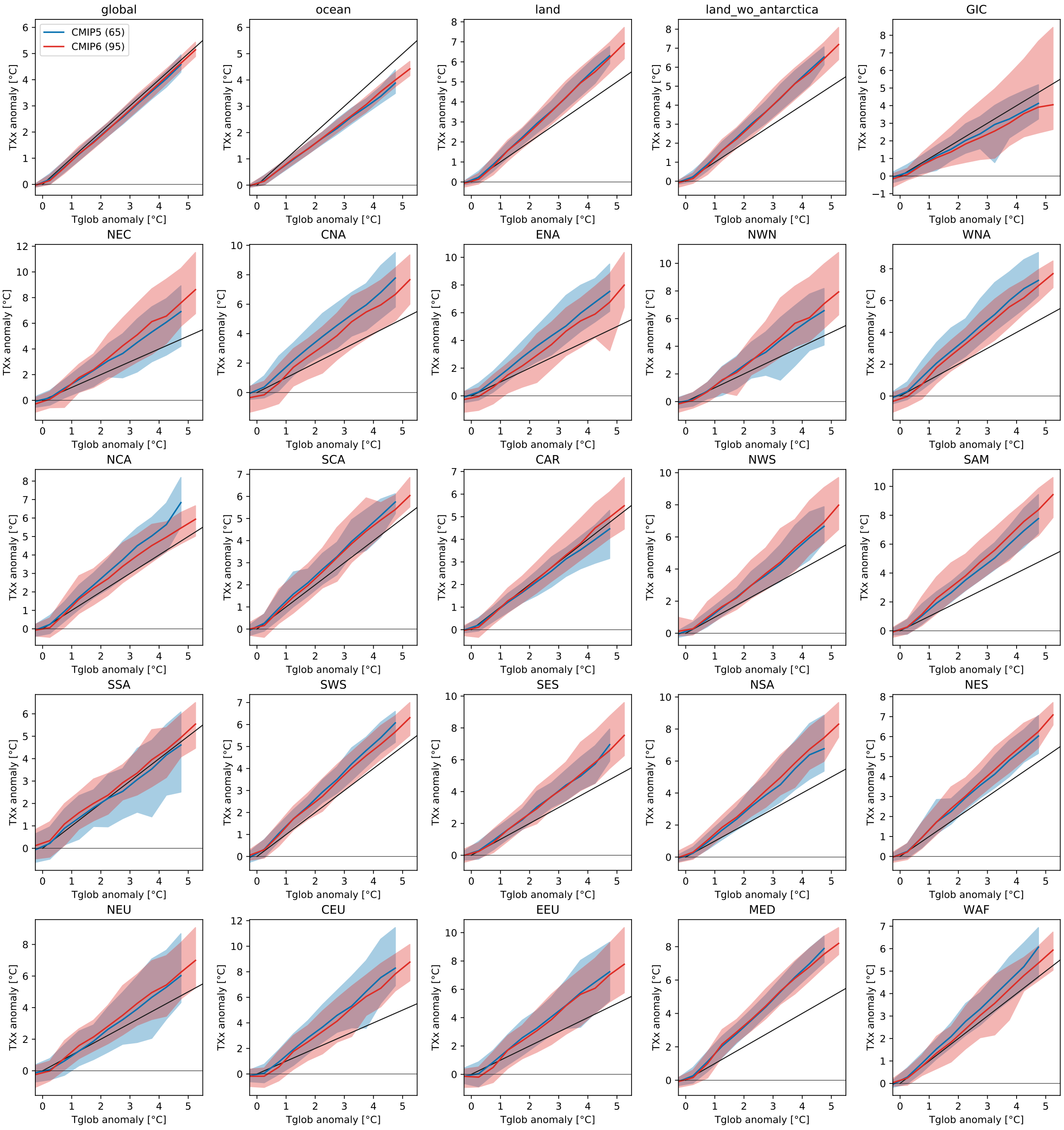

Figure S10: As Figure S8 but for TXx.

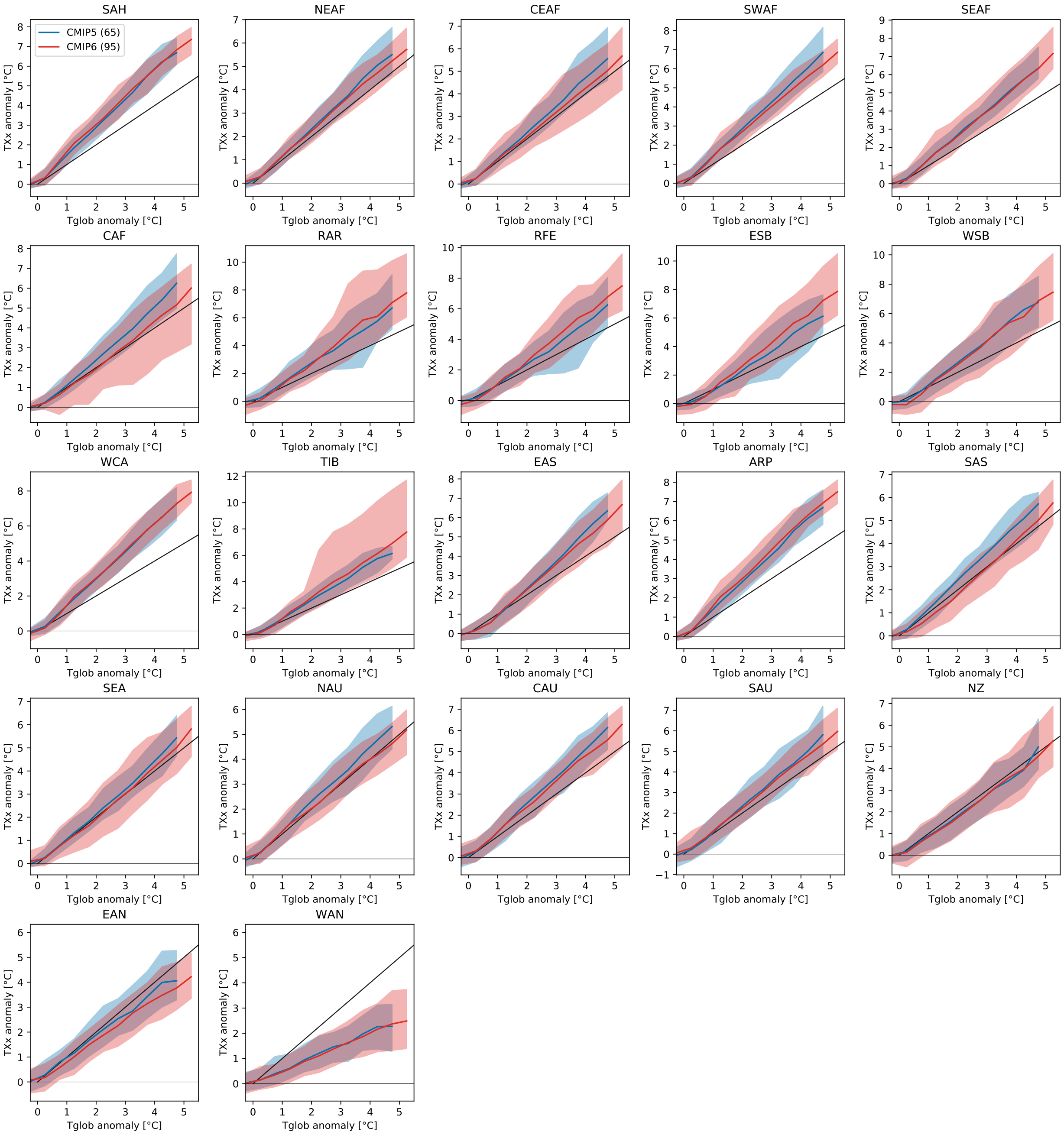

Figure S11: As Figure S10 for regions 22 to 43.

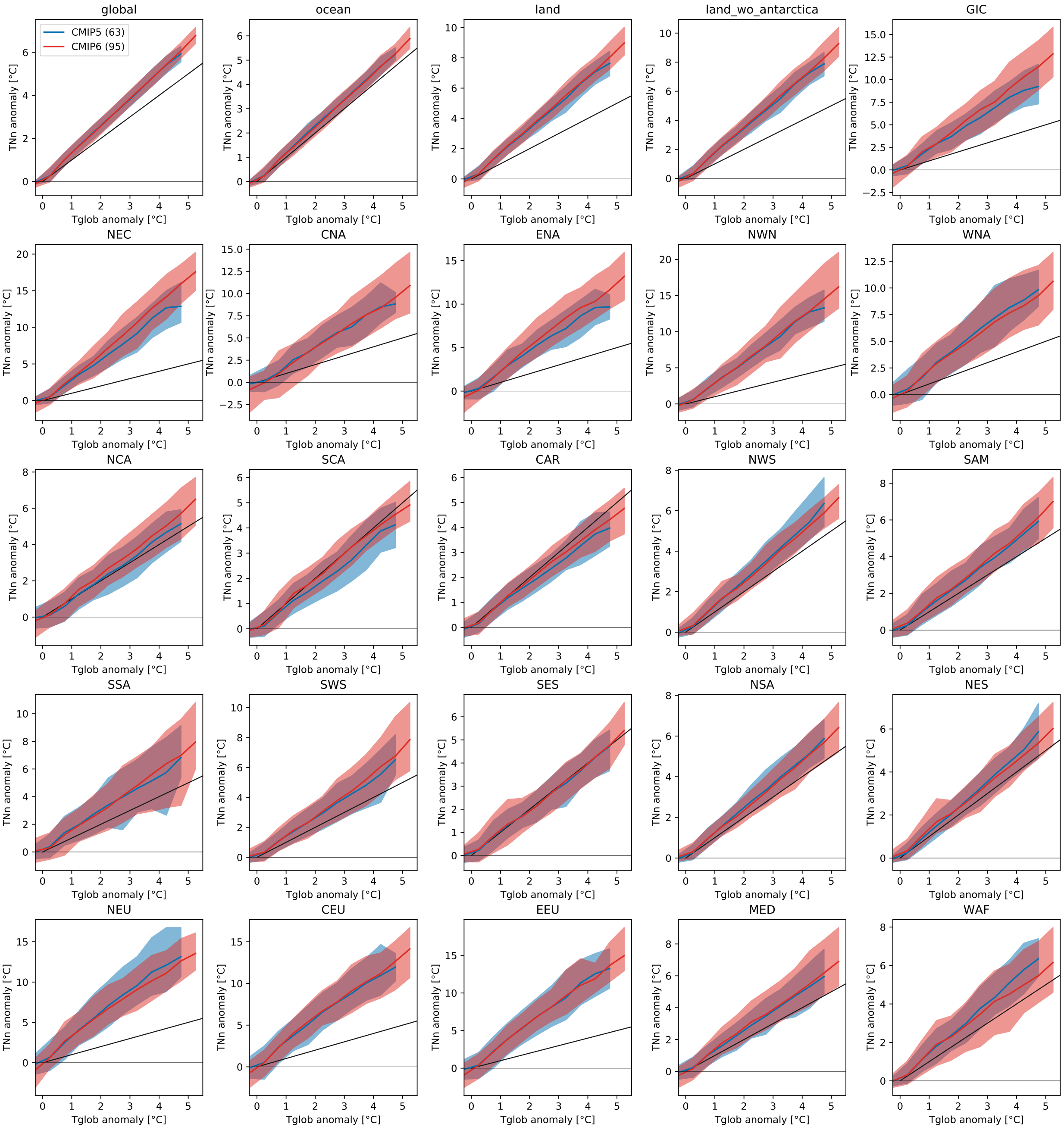

Figure S12: As Figure S8 but for TNn.

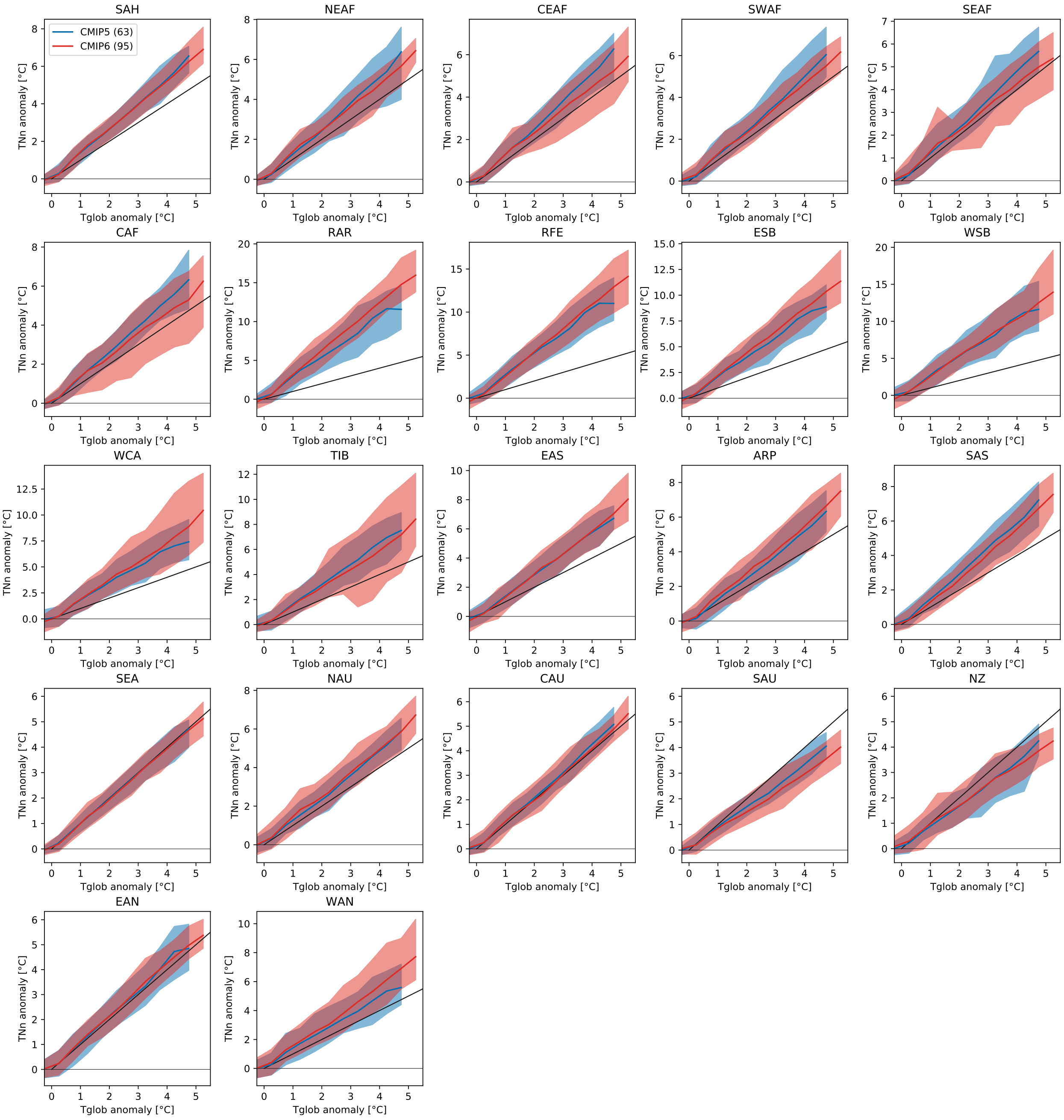

Figure S13: As Figure S12 for regions 22 to 43.

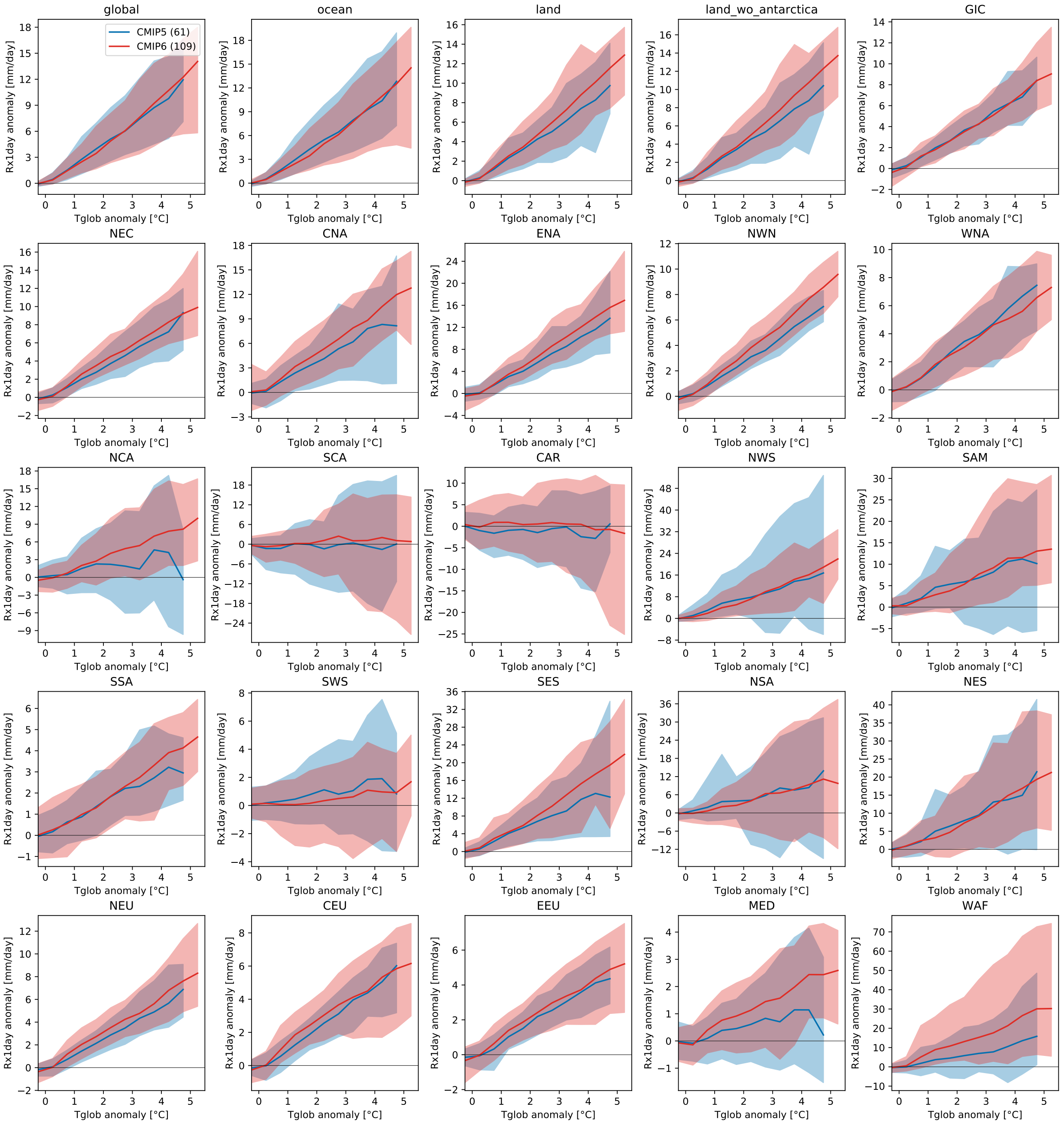

Figure S14: As Figure S8 but for Rx1day.

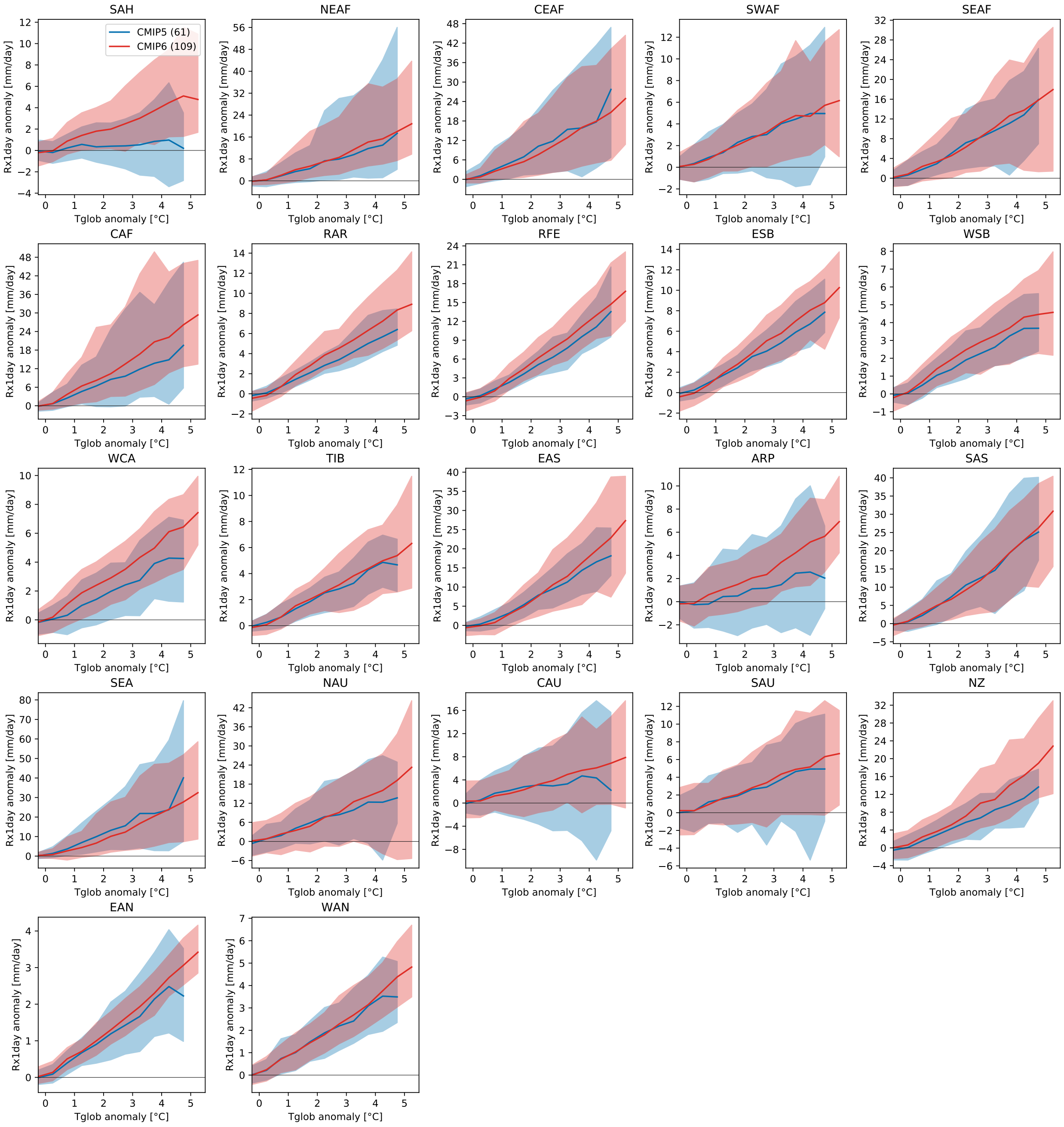

Figure S15: As Figure S14 for regions 22 to 43.

Additional tables

Table S1: Range (minimum and maximum; 5th and 95th percentile) and central values (mean and median) of TXx at a global mean warming of 1.5°C for CMIP5 and CMIP6 ensemble.

	CMIP5						CMIP6					
region	min [°C]	p5 [°C]	mean [°C]	med. [°C]	p95 [°C]	max [°C]	min [°C]	p5 [°C]	mean [°C]	med. [°C]	p95 [°C]	max [°C]
global	1.29	1.31	1.40	1.39	1.48	1.52	1.14	1.26	1.40	1.39	1.55	1.59
ocean	1.08	1.12	1.19	1.18	1.32	1.35	1.03	1.12	1.21	1.21	1.31	1.33
land	1.66	1.71	1.92	1.87	2.25	2.31	1.30	1.43	1.88	1.86	2.32	2.39
land_wo_antarctica	1.65	1.75	1.98	1.92	2.32	2.42	1.38	1.51	1.94	1.93	2.41	2.52
ARP	1.58	1.63	2.14	2.16	2.67	2.75	1.55	1.90	2.34	2.27	3.10	3.42
CAF	1.10	1.25	1.77	1.71	2.36	2.52	-0.36	0.26	1.43	1.60	2.23	2.70
CAR	0.76	1.05	1.45	1.47	1.78	1.91	0.60	1.08	1.51	1.47	2.05	2.16
CAU	1.19	1.30	1.93	1.97	2.43	2.73	0.90	1.21	1.83	1.78	2.42	3.07
CEAF	1.19	1.44	1.73	1.67	2.11	2.50	0.85	1.04	1.62	1.61	2.57	3.14
CEU	0.91	1.34	2.47	2.36	3.66	5.06	0.36	0.72	2.15	2.20	3.40	3.66
CNA	1.18	1.47	2.58	2.58	4.01	4.36	0.31	0.82	2.07	2.22	3.10	3.55
EAN	0.63	0.89	1.42	1.41	1.93	2.44	0.20	0.51	1.22	1.24	1.89	1.96
EAS	0.59	0.88	1.59	1.67	2.23	2.46	0.36	0.72	1.63	1.67	2.37	2.66
EEU	0.49	1.20	2.28	2.16	3.28	4.19	0.10	0.66	2.05	2.15	3.16	3.35
ENA	1.36	1.51	2.34	2.31	3.33	3.43	-0.94	0.31	1.84	1.98	2.88	3.22
ESB	0.66	0.79	1.58	1.54	2.52	3.02	0.37	0.64	1.91	2.02	2.92	3.32
GIC	0.28	0.72	1.35	1.39	1.99	2.16	0.39	0.49	1.25	1.19	2.66	2.82
MED	1.79	1.97	2.44	2.42	2.91	3.10	0.77	1.88	2.52	2.48	3.28	3.99
NAU	1.16	1.35	1.76	1.76	2.26	2.39	0.58	1.01	1.57	1.55	2.21	2.62
NCA	1.05	1.60	2.04	2.07	2.51	2.95	0.99	1.15	1.89	1.78	2.70	3.54
NEAF	1.15	1.40	1.75	1.74	2.17	2.31	1.35	1.42	1.73	1.67	2.23	2.86
NEC	0.79	0.91	1.96	2.00	2.80	3.01	0.43	0.92	2.11	2.13	2.97	3.46
NES	1.34	1.51	2.01	1.99	2.55	4.08	1.32	1.53	2.10	1.98	3.02	3.39
NEU	0.19	0.60	1.60	1.49	2.63	3.31	0.45	1.01	1.85	1.80	2.66	3.84
NSA	1.24	1.50	2.08	2.05	2.65	2.99	1.56	1.71	2.23	2.13	3.00	3.28
NWN	0.38	0.71	1.85	1.90	2.75	3.24	0.28	0.84	1.93	1.92	2.94	3.22
NWS	1.03	1.50	1.95	1.94	2.54	2.81	1.23	1.40	2.04	1.92	3.60	4.35
NZ	0.40	0.70	1.40	1.45	1.98	2.30	0.10	0.46	1.33	1.35	2.14	2.60
RAR	0.32	1.17	2.04	2.09	3.05	3.26	0.73	1.05	1.93	2.00	2.76	3.11
RFE	0.73	0.92	1.68	1.57	2.68	2.83	0.64	0.92	1.84	1.92	2.60	2.69
SAH	1.68	1.78	2.20	2.16	2.60	2.68	1.33	1.97	2.39	2.39	3.07	3.47
SAM	1.40	1.68	2.31	2.28	3.07	3.49	1.19	1.62	2.63	2.56	4.30	4.84
SAS	0.76	1.12	1.81	1.81	2.20	2.64	-0.27	0.30	1.20	1.25	1.91	2.12
SAU	0.93	1.07	1.78	1.80	2.53	2.67	0.61	1.01	1.69	1.67	2.38	3.27
SCA	1.26	1.37	1.86	1.80	2.41	3.45	-0.17	1.01	1.75	1.70	2.84	3.21
SEA	1.19	1.30	1.61	1.53	2.22	2.67	0.01	0.60	1.51	1.40	2.69	3.56
SEAF	1.49	1.76	2.07	2.01	2.43	2.94	1.21	1.37	2.01	1.86	3.02	3.79
SES	1.38	1.45	2.01	1.94	2.62	2.91	1.17	1.43	2.04	1.96	3.00	3.43
SSA	0.41	0.82	1.60	1.56	2.46	3.19	0.78	1.08	1.85	1.80	2.91	3.58
SWAF	1.60	1.82	2.19	2.16	2.58	2.89	1.43	1.70	2.10	2.05	2.92	3.66
SWS	1.58	1.69	2.00	1.99	2.29	2.55	1.39	1.47	1.97	1.93	2.52	3.22
TIB	1.20	1.43	1.87	1.85	2.39	2.56	0.99	1.31	2.09	1.91	4.13	5.34
WAF	1.35	1.41	1.85	1.86	2.26	2.33	0.56	0.89	1.60	1.58	2.38	3.16
WAN	0.13	0.37	0.78	0.74	1.26	1.68	-0.09	0.21	0.71	0.65	1.29	1.32
WCA	1.43	1.70	2.22	2.21	2.83	2.92	1.11	1.70	2.30	2.35	3.02	3.51
WNA	1.43	1.67	2.42	2.30	3.58	4.74	0.94	1.30	2.21	2.24	3.04	3.40
WSB	0.45	0.92	1.93	1.84	2.84	3.33	-0.37	0.63	1.82	2.01	2.75	3.01

Table S2: Global mean temperature of individual CMIP5 models at a multi-model mean warming of 1.5°C, and minimum, 5th percentile, mean, median, 95th percentile, and maximum global warming for the different scenarios individually and pooled.

	RCP2.6	RCP4.5	RCP6.0	RCP8.5	pooled
ACCESS1-0	–	1.46	–	1.42	–
ACCESS1-3	–	1.28	–	1.30	–
BNU-ESM	2.09	2.18	–	2.09	–
CCSM4	1.72	1.73	1.78	1.84	–
CESM1-BGC	–	1.68	–	1.73	–
CESM1-CAM5	1.51	1.47	1.44	1.42	–
CESM1-CAM5-1-FV2	–	1.30	–	1.23	–
CMCC-CESM	–	–	–	1.03	–
CMCC-CM	–	1.35	–	1.31	–
CMCC-CMS	–	1.34	–	1.29	–
CNRM-CM5	1.29	1.29	–	1.31	–
CSIRO-Mk3-6-0	1.37	1.27	1.06	1.15	–
CanESM2	1.92	1.91	–	1.94	–
EC-EARTH	1.57	1.65	–	1.65	–
FGOALS-g2	1.09	1.29	–	1.31	–
FGOALS-s2	–	–	–	2.38	–
FIO-ESM	1.38	1.40	1.51	1.41	–
GISS-E2-H	1.51	1.65	1.63	1.63	–
GISS-E2-H-CC	–	1.73	–	1.69	–
GISS-E2-R	1.17	1.24	1.30	1.27	–
GISS-E2-R-CC	–	1.36	–	1.28	–
IPSL-CM5A-LR	1.92	1.97	1.99	1.95	–
IPSL-CM5A-MR	1.70	1.88	1.78	1.80	–
IPSL-CM5B-LR	–	1.56	–	1.56	–
MPI-ESM-LR	1.61	1.64	–	1.68	–
MPI-ESM-MR	1.56	1.59	–	1.64	–
MRI-CGCM3	0.95	1.05	1.03	0.98	–
NorESM1-M	1.22	1.26	1.20	1.24	–
NorESM1-ME	1.24	1.22	1.23	1.27	–
bcc-csm1-1	1.64	1.69	1.67	1.63	–
bcc-csm1-1-m	1.87	1.90	1.94	1.89	–
inmcm4	–	1.02	–	1.07	–
min	0.95	1.02	1.03	0.98	0.95
p5	1.08	1.12	1.05	1.05	1.04
mean	1.52	1.51	1.50	1.51	1.51
med.	1.53	1.47	1.51	1.42	1.51
p95	1.93	1.94	1.96	2.02	1.97
max	2.09	2.18	1.99	2.38	2.38

Table S3: As Table S2 but for CMIP6.

	SSP1-1.9	SSP1-2.6	SSP2-4.5	SSP3-7.0	SSP5-8.5	pooled
ACCESS-CM2	–	1.48	1.45	1.51	1.51	–
ACCESS-ESM1-5	–	1.37	1.41	1.36	1.41	–
BCC-CSM2-MR	–	1.25	1.23	1.33	1.31	–
CAMS-CSM1-0	1.05	1.00	0.98	1.06	1.09	–
CESM2	–	1.54	1.52	1.59	1.57	–
CESM2-WACCM	–	1.69	1.58	1.50	1.67	–
CNRM-CM6-1	–	1.48	1.40	1.37	1.42	–
CNRM-CM6-1-HR	–	1.87	1.77	1.82	1.80	–
CNRM-ESM2-1	1.28	1.25	1.27	1.23	1.29	–
CanESM5	2.12	2.04	2.14	2.25	2.15	–
CanESM5-CanOE	–	2.23	2.31	2.50	2.35	–
EC-Earth3	1.76	1.60	1.62	1.65	1.55	–
EC-Earth3-Veg	2.00	1.93	1.89	1.87	1.96	–
FGOALS-f3-L	–	1.69	1.67	1.71	1.66	–
FGOALS-g3	–	1.32	1.41	1.51	1.46	–
FIO-ESM-2-0	–	1.81	1.80	–	1.83	–
GFDL-CM4	–	–	1.36	–	1.36	–
GFDL-ESM4	1.20	1.16	1.20	1.14	1.11	–
GISS-E2-1-G	–	1.82	1.80	1.81	1.80	–
HadGEM3-GC31-LL	–	1.71	1.76	–	1.78	–
INM-CM4-8	–	1.23	1.28	1.28	1.36	–
INM-CM5-0	–	1.31	1.26	1.36	1.32	–
IPSL-CM6A-LR	1.79	1.72	1.75	1.72	1.70	–
KACE-1-0-G	–	2.12	2.17	2.19	2.12	–
MCM-UA-1-0	–	1.57	1.57	1.58	1.61	–
MIROC-ES2L	1.26	1.18	1.17	1.22	1.18	–
MIROC6	1.09	1.06	1.07	1.06	1.06	–
MPI-ESM1-2-HR	–	1.32	1.32	1.38	1.33	–
MPI-ESM1-2-LR	–	1.29	1.28	1.31	1.26	–
MRI-ESM2-0	1.47	1.42	1.38	1.41	1.49	–
NESM3	–	1.65	1.57	–	1.67	–
NorESM2-LM	–	0.98	0.96	0.96	1.04	–
NorESM2-MM	–	1.01	–	0.94	1.02	–
UKESM1-0-LL	1.69	1.66	1.65	1.79	1.65	–
min	1.05	0.98	0.96	0.94	1.02	0.94
p5	1.07	1.01	1.03	1.01	1.05	1.02
mean	1.52	1.51	1.51	1.51	1.53	1.52
med.	1.47	1.48	1.45	1.45	1.50	1.48
p95	2.06	2.07	2.15	2.23	2.14	2.15
max	2.12	2.23	2.31	2.50	2.35	2.50

Table S4: As Table S2 but for a multi model mean warming of 4.0°C.

	RCP2.6	RCP4.5	RCP6.0	RCP8.5	pooled
ACCESS1-0	–	–	–	4.21	–
ACCESS1-3	–	–	–	4.12	–
BNU-ESM	–	–	–	5.08	–
CCSM4	–	–	–	4.33	–
CESM1-BGC	–	–	–	4.15	–
CESM1-CAM5	–	–	–	4.33	–
CESM1-CAM5-1-FV2	–	–	–	4.17	–
CMCC-CESM	–	–	–	3.89	–
CMCC-CM	–	–	–	4.32	–
CMCC-CMS	–	–	–	4.40	–
CNRM-CM5	–	–	–	3.82	–
CSIRO-Mk3-6-0	–	–	–	4.07	–
CanESM2	–	–	–	4.94	–
EC-EARTH	–	–	–	4.06	–
FGOALS-g2	–	–	–	3.31	–
FGOALS-s2	–	–	–	5.72	–
FIO-ESM	–	–	–	3.88	–
GISS-E2-H	–	–	–	3.60	–
GISS-E2-H-CC	–	–	–	3.62	–
GISS-E2-R	–	–	–	2.94	–
GISS-E2-R-CC	–	–	–	3.01	–
IPSL-CM5A-LR	–	–	–	5.10	–
IPSL-CM5A-MR	–	–	–	4.93	–
IPSL-CM5B-LR	–	–	–	3.95	–
MPI-ESM-LR	–	–	–	4.13	–
MPI-ESM-MR	–	–	–	4.06	–
MRI-CGCM3	–	–	–	3.33	–
NorESM1-M	–	–	–	3.45	–
NorESM1-ME	–	–	–	3.64	–
bcc-csm1-1	–	–	–	4.02	–
bcc-csm1-1-m	–	–	–	3.94	–
inmcm4	–	–	–	3.01	–
min	–	–	–	2.94	2.94
p5	–	–	–	3.01	3.01
mean	–	–	–	4.05	4.05
med.	–	–	–	4.06	4.06
p95	–	–	–	5.09	5.09
max	–	–	–	5.72	5.72

Table S5: As Table S5 but for CMIP6.

	SSP1-1.9	SSP1-2.6	SSP2-4.5	SSP3-7.0	SSP5-8.5	pooled
ACCESS-CM2	–	–	–	4.51	4.42	–
ACCESS-ESM1-5	–	–	–	3.96	3.91	–
BCC-CSM2-MR	–	–	–	3.66	3.47	–
CAMS-CSM1-0	–	–	–	2.74	2.62	–
CESM2	–	–	–	4.31	4.52	–
CESM2-WACCM	–	–	–	4.29	4.64	–
CNRM-CM6-1	–	–	–	4.42	4.38	–
CNRM-CM6-1-HR	–	–	–	4.75	4.73	–
CNRM-ESM2-1	–	–	–	4.07	3.85	–
CanESM5	–	–	–	5.89	5.80	–
CanESM5-CanOE	–	–	–	6.07	5.94	–
EC-Earth3	–	–	–	4.34	4.24	–
EC-Earth3-Veg	–	–	–	4.79	4.62	–
FGOALS-f3-L	–	–	–	3.91	3.94	–
FGOALS-g3	–	–	–	3.26	3.11	–
FIO-ESM-2-0	–	–	–	–	4.46	–
GFDL-CM4	–	–	–	–	3.88	–
GFDL-ESM4	–	–	–	3.26	3.05	–
GISS-E2-1-G	–	–	–	3.91	3.92	–
HadGEM3-GC31-LL	–	–	–	–	5.01	–
INM-CM4-8	–	–	–	3.24	3.25	–
INM-CM5-0	–	–	–	3.20	3.11	–
IPSL-CM6A-LR	–	–	–	4.86	4.83	–
KACE-1-0-G	–	–	–	4.86	4.87	–
MCM-UA-1-0	–	–	–	3.90	4.02	–
MIROC-ES2L	–	–	–	3.21	3.27	–
MIROC6	–	–	–	2.98	3.04	–
MPI-ESM1-2-HR	–	–	–	3.38	3.14	–
MPI-ESM1-2-LR	–	–	–	3.42	3.27	–
MRI-ESM2-0	–	–	–	3.61	3.69	–
NESM3	–	–	–	–	4.28	–
NorESM2-LM	–	–	–	2.87	2.97	–
NorESM2-MM	–	–	–	3.01	3.02	–
UKESM1-0-LL	–	–	–	5.47	5.34	–
min	–	–	–	2.74	2.62	2.62
p5	–	–	–	2.92	3.01	2.97
mean	–	–	–	4.01	4.02	4.01
med.	–	–	–	3.91	3.93	3.92
p95	–	–	–	5.70	5.50	5.75
max	–	–	–	6.07	5.94	6.07

References

Iturbide, M., J. Gutiérrez, L. Alves, J. Bedia, E. Cimadevilla, A. Cofino, R. Cerezo-Mota, A. Di Luca, S. Faria, I. Gorodetskaya, M. Hauser, S. Herrera, H. Hewitt, K. Hennessy, R. Jones, S. Krakovska, R. Manzanas, D. Martínez-Castro, G. Narisma, I. Nurhati, I. Pinto, S. Seneviratne, B. van den Hurk, and C. Vera (2020). "An update of IPCC physical climate reference regions for subcontinental analysis of climate model data: Definition and aggregated datasets". In: *Earth System Science Data Discussions*. in review. DOI: 10.5194/essd-2019-258 (cit. on p. 2).
